# Deep learning-enabled breast cancer endocrine response determination from H&E staining based on ESR1 signaling activity

**DOI:** 10.1038/s41598-023-48830-x

**Published:** 2023-12-05

**Authors:** Chun Wai Ng, Kwong-Kwok Wong

**Affiliations:** Department of Gynecologic Oncology and Reproductive Medicine, Unit 1362, The University of Texas MD Anderson Cancer Center, 1515 Holcombe Blvd, Houston, TX 77030 USA

**Keywords:** Breast cancer, Cancer screening, Cancer genomics

## Abstract

Estrogen receptor (ER) positivity by immunohistochemistry has long been a main selection criterium for breast cancer patients to be treated with endocrine therapy. However, ER positivity might not directly correlate with activated ER signaling activity, which is a better predictor for endocrine therapy responsiveness. In this study, we investigated if a deep learning method using whole-slide H&E-stained images could predict ER signaling activity. First, ER signaling activity score was determined using RNAseq data available from each of the 1082 breast cancer samples in the TCGA Pan-Cancer dataset based on the Hallmark Estrogen Response Early gene set from the Molecular Signature Database (MSigDB). Then the processed H&E-stained images and ER signaling activity scores from a training cohort were fed into ResNet101 with three additional fully connected layers to generate a predicted ER activity score. The trained models were subsequently applied to an independent testing cohort. The result demonstrated that ER + /HER2- breast cancer patients with a higher predicted ER activity score had longer progression-free survival (*p* = 0.0368) than those with lower predicted ER activity score. In conclusion, a convolutional deep neural network can predict prognosis and endocrine therapy response in breast cancer patients based on whole-slide H&E-stained images. The trained models were found to robustly predict the prognosis of ER + /HER2- patients. This information is valuable for patient management, as it does not require RNA-seq or microarray data analyses. Thus, these models can reduce the cost of the diagnosis workflow if such information is required.

## Introduction

Breast cancers can be categorized into three major subtypes on the basis of the hormone receptors: estrogen receptor (ER) positivity, progesterone receptor (PR) positivity and human epidermal growth factor 2 (HER2, also known as ERBB2) positivity^1–3^. ER + /HER2- breast cancer is the most common subtype, constituting about 70% of the cases; the ER-/HER2 + subtype constitutes about 15–20%, and the ER-/PR-/HER2- subtype (triple-negative breast cancer [TNBC]) constitutes about 15%^4,5^. ER + breast cancers depend on ER signaling for proliferation, while HER2 + breast cancers depend on the HER2 signaling pathway.

Endocrine therapy reduces the recurrence rate of ER + breast cancers by about 50% and improves survival time by targeting ER and thus its downstream signaling. The most commonly used endocrine therapy drugs are tamoxifen, letrozole, and fulvestrant^6^. Although these drugs have relatively mild side effects, mainly post-menopausal symptoms, about 40% of patients do not complete the full 5-year treatment^7^; there is a need to identify patients who will experience a response to endocrine therapy to improve quality of life for the patients by avoiding unnecessary treatment.

The coupling of ER expression and ER pathway activity is essential for cell proliferation through ER signaling and endocrine therapy responsiveness^8–10^. Since ER + /HER2- cancers are commonly treated with endocrine therapy, recent study has found that ER pathway activity was significantly associated with survival duration in patients with ER + /HER2- breast cancer^9^. This implies that ER expression and elevated levels of ER pathway activity are related to endocrine therapy responsiveness. The ability to predict the efficacy of endocrine therapy in ER + /HER2- patients would facilitate the decision-making process for both physicians and patients. However, unlike hematoxylin-and-eosin (H&E) staining and ER/HER2/PR immunohistochemistry staining of tumor samples, the determination of ER pathway activity requires transcriptomic analyses that are not part of standard diagnostic procedures; thus, these data are usually unavailable.

Deep learning models for the prediction of ER/PR/HER2 status with H&E whole-slide images have been proposed in different studies^11,12^. However, there is also a need to predict ER signaling activity to determine prognosis and endocrine therapy effectiveness as no such study has been done. In this study, we determined whether a convolutional deep neural network and whole-slide H&E-stained images of The Cancer Genome Atlas (TCGA) breast cancer tumor samples could be used to predict ER signaling pathway activity to determine prognosis in patients with ER + /HER2- breast cancer. We measured ER pathway activity using the Early Estrogen Response Enrichment Score (EERES), determined by a Gene Set Variance Analysis (GSVA) with the Molecular Signature Database (MSigDB) hallmark Estrogen Response Early gene set^13–15^. We hypothesized that the predicted ER activity score from the deep learning model could determine endocrine therapy response in ER + /HER2- breast cancer patients. In line with our hypothesis, our results demonstrated that the predicted ER activity scores from whole-slide H&E-stained images were significantly associated with progression-free survival (PFS) in ER + /HER- breast cancer patients.

## Results

### ER + /HER2- breast cancer patient survival based on the EERES

**Figure 1:**
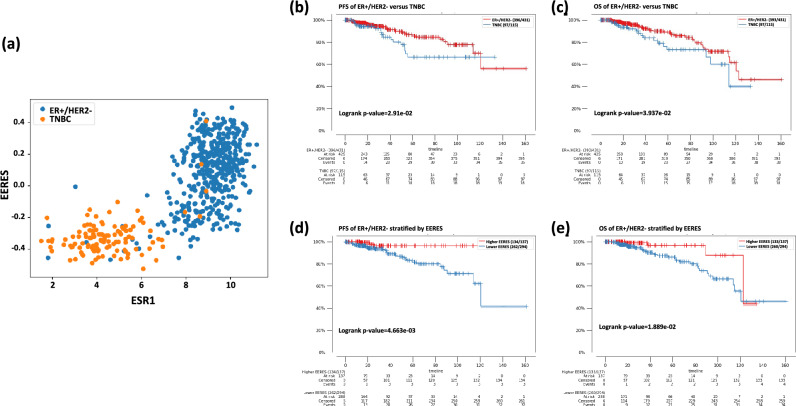
Survival of 1082 patients with ER + /HER2- breast cancer based on EERES, as determined by GSVA using the Hallmark Estrogen Response Early MSigDB gene set. (**a**) Scatter plot of ESR1 gene expression and EERES of ER + /HER2- (blue) breast cancer and TNBC (yellow) tumor samples. (**b**) PFS and (**c**) OS durations of ER + /HER2- breast cancer and TNBC tumor samples, tested with log-rank tests and shown as Kaplan–Meier curves. ER + /HER2- breast cancer tumor samples were stratified into higher and lower EERES, as described in the Methods, and their (**d**) PFS and (**e**) OS durations are shown as Kaplan–Meier curves.

The EERES of level 3 gene expression data (RNAseq) on 1082 TCGA Pan-Cancer breast cancer patient tumor samples from cBioPortal were determined by GSVA with the Estrogen Response Early gene set from MsigDB as described in the method section. The determined EERES scores for each sample are provided in Supplemental Table S1. The correlation between ESR1 gene expression and EERES for ER + /HER2- breast cancer and TNBC tumor samples are shown in Fig. 1a. The PFS and overall survival (OS) durations of ER + /HER2- and TNBC breast cancer patients are shown in Fig. 1b and c, respectively. The OS durations (*p* = 0.0394) of ER + /HER2- breast cancer patients were significantly longer than those of TNBC patients.

We next determined the association between the EERES and ER + /HER2- breast cancer patient survival (Fig. 1d and e, respectively). The PFS (*p* = 0.00466) and OS (*p* = 0.0189) durations were significantly longer for patients with an EERES > 0.2. As expected, the PFS/OS durations of TNBC patients were not statistically significant according to the EERES (data not shown).

### Model training and evaluation

**Figure 2:**
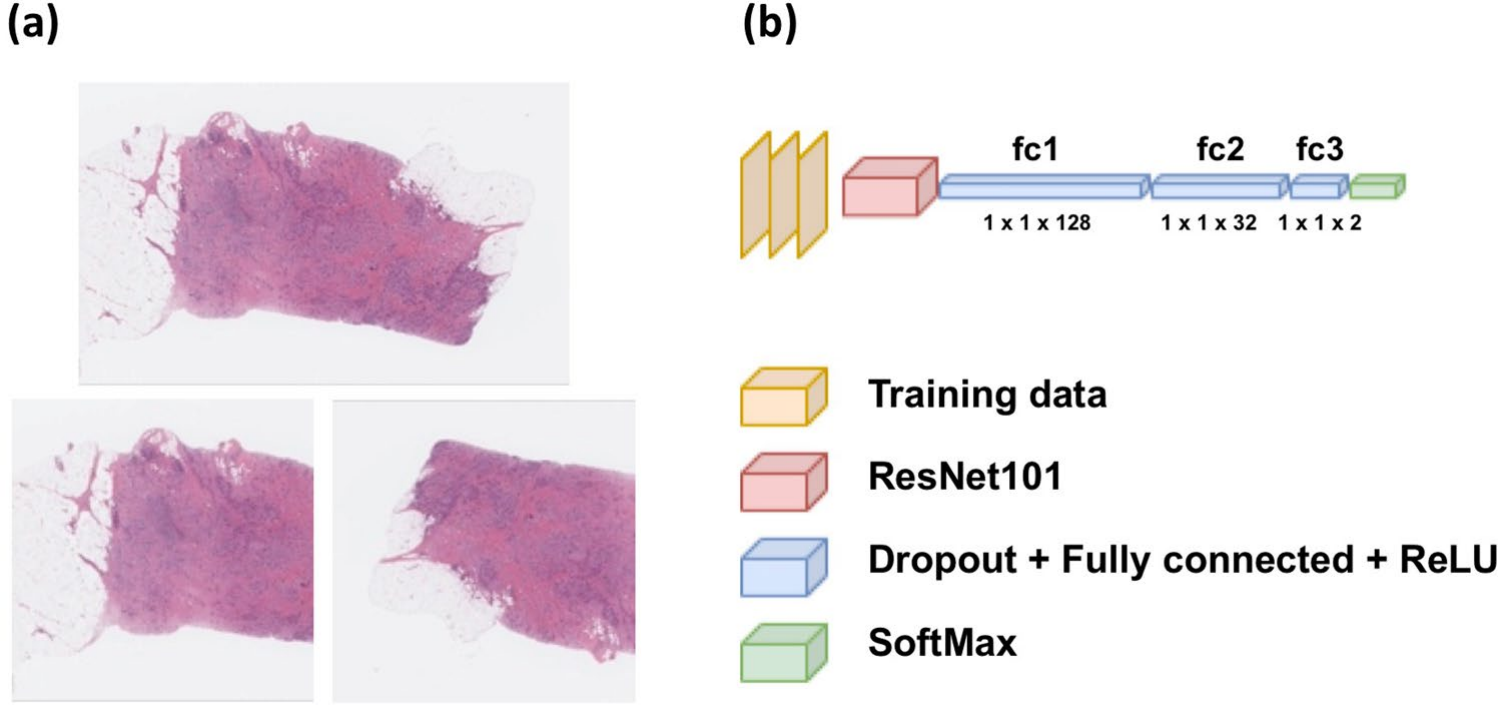
Overview of model architecture. The H&E-stained whole-slide images of breast tumors were annotated and cropped before training and evaluation. (**a**) Examples of the original (top) and training and evaluation images (bottom) after cropping and augmentation. (**b**) Pre-trained ResNet101, which outputs 1000 perceptrons, was connected with three additional fully connected layers with ReLU activation and SoftMax activation. The models were trained and evaluated with the cropped whole-slide images, as shown in (**a**).

Examples of the image preparation and model architecture are shown in Fig. 2. The training data was labeled 0 or 1 according to the EERES at the cut-off quantile of 0.5 (label 0 if EERES ≤ − 0.0376; label 1 if EERES > − 0.0376). The metadata for the 1077 images are provided in Supplemental Table S2. The model was trained for 30 epochs.

**Figure 3:**
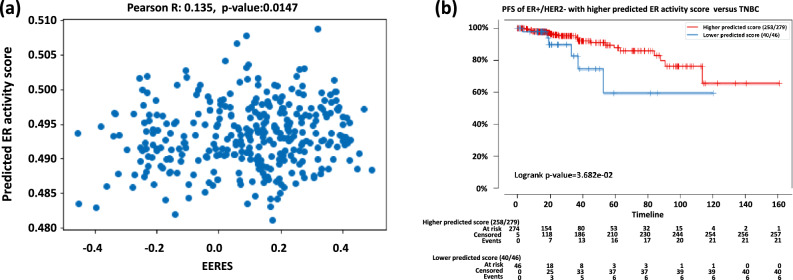
Model performance with testing data. After training, we evaluated the model with unseen testing patient cohort from Broad Institute of MIT (n = 812). The predicted scores for each sample were output by the trained model, and (**a**) the scatter plot showing the correlation between the EERES and the predicted ER activity score of ER + /HER2- patients is shown. The best-predicted score threshold was used to stratify patients as higher or lower predicted ER activity score groups and their (**b**) PFS is shown as Kaplan–Meier curves for the ER + /HER2- testing cohort. The ER + /HER2- patients with higher and lower predicted ER activity score were compared for their survival using Kaplan–Meier curve.

The predicted ER activity scores of the testing cohort (n = 811, one sample without known hormonal receptor status was ignored) were determined by the trained model and are provided in Supplemental Table S3. The correlation of the predicted ER activity scores of ER + /HER2- with the actual EERES was determined with Pearson correlation test and the scatter plot with the statistics are shown in Fig. 3a (r = 0.135, *p* = 0.0147). The ER + /HER2- patients with higher and lower predicted ER activity score were compared for their survival using Kaplan–Meier curve (Fig. 3b). We found that ER + /HER2- patients with higher predicted score (quantile 0.2, predicted ER activity score threshold at 0.48964) had significantly longer response time (*p* = 0. 0368), indicating that higher predicted ER activity score was related to a better endocrine therapy response.

**Figure 4:**
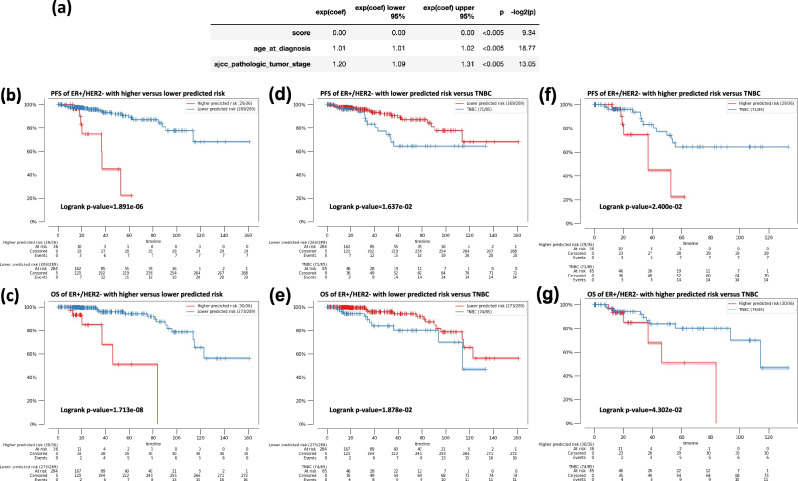
Survival analyses of ER + /HER2- breast cancer patients with lower and higher predicted risk score from CoxPH models. (**a**) The multivariate correlation analysis of the predicted ER activity score, diagnostic age, and stage of testing samples with PFS. Exp(coef) is the hazard ratio (HR), The upper and lower 95% were the 95% confidence interval. P corresponds to the p-value. A predicted risk score for each testing sample in the testing cohort was generated using model fitted using ER activity score, clinical data and PFS of the training cohort. ER + /HER2- patients were assigned with a higher and lower predicted risk for PFS using a quantile 0.9 as cut-off. (**b**) Comparison of progression free survival (PFS) of predicted higher risk ER + /HER2- patients with lower risk ER + /HER2- patients. (**c**) Comparison of overall survival (OS) of predicted higher risk ER + /HER2- patients with lower risk ER + /HER2- patients. (**d**) Comparison of PFS of predicted lower risk ER + /HER2- patients with triple negative breast cancer (TNBC) patients. (**e**) Comparison of OS of predicted lower risk ER + /HER2- patients with TNBC patients. (**f**) Comparison of PFS of predicted higher risk ER + /HER2- patients with TNBC patients. (**g**) Comparison of OS of predicted higher risk ER + /HER2- patients with TNBC patients.

Moreover, to consider other clinical data (diagnostic age and stage) might impact treatment response (PFS), multivariant analysis of predicted ER activity score, age and stage of the testing samples were fitted into a cox proportional hazard (CoxPH) model with PFS duration. As shown in Fig. 4a, the predicted ER activity had an extreme low hazard ratio (HR) (HR < 0.001, *p* < 0.005), which implies a much lower risk of tumor progression with a higher predicted ER activity score. On the other hand, a higher stage (HR = 1.2, *p* < 0.005) and an older diagnostic age (HR = 1.01, *p* < 0.005) might have an increased risk of tumor progression. Thus, we fitted a CoxPH using data including predicted ER activity score, diagnostic age, and stage with PFS duration data from the training cohort to generate a risk score and applied the model to predict a risk score for the testing cohort. The resulting Kaplan–Meier curves for the PFS and OS duration between high and low predicted risk ER + /HER2- patients are shown in Fig. 4b and c, respectively. The PFS (*p* = 6.38e-6) and OS (*p* = 4.73e-10) durations were significantly longer for patients with a lower predicted risk at quantile 0.9.

The predicted risks of the ER + /HER2- patients were further evaluated by comparing their survival durations with those of TNBC patients (Fig. 4d–g). The PFS (*p* = 0.0206) and OS (*p* = 0.0178) durations of the testing ER + /HER2- patients with lower predicted risks were significantly longer than those of TNBC patients. On the other hand, the PFS and OS (*p* = 0.0152 and *p* = 0.0177, respectively) of ER + /HER2- patients with higher predicted risks had even shorter survival than TNBC patients.

## Discussion

In this study, we presented a novel model based on ESR1 signaling activity score that can predict the prognosis and endocrine therapy response of ER + /HER2- breast cancer patients from whole-slide tumor H&E staining using a convolution deep neural network. The trained models were found to robustly predict the prognosis of ER + /HER2- patients; this information is valuable for patient management, as it does not require RNA-seq or microarray data analyses. Thus, these models can reduce the cost of the diagnosis workflow if such information is required. Using a CoxPH model based on the predicted ER activity score, age and stage, we were able to identify ER + /HER2- breast cancer patients from a testing cohort with high risk of progressive disease and even had poorer survival than TNBC patients. These high-risk patients could be spared from unnecessary endocrine therapy.

The model demonstrated its ability to distinguish patients with different prognoses according to the level of the predicted ER activity score from whole-slide tumor H&E staining for ER + /HER2- disease. In addition, the statistical difference between patients with ER + /HER2- breast cancer with a higher predicted ER activity score (but not those with a lower predicted ER activity score) and patients with TNBC confirmed that our model is predictive of endocrine therapy response in those with ER + /HER2- disease but not in those without ESR1 expression. This model could be developed as a medical device to evaluate treatment but will need further development^16^. Moreover, as EERES is related to the lower American Joint Committee on Cancer stage and the lower pathological grade, this model could also supplement this information^9^.

Other models have been extensively developed using convolutional neural networks and H&E images to identify mutation and receptor expression status, such as for the prediction of the expression of hormonal receptors, PD-L1, and BRCA mutations^11,12,17–20^. Cancer prognosis can also be predicted using histological information from tumor H&E images, together with other omics data^21^. However, predicting signaling activity to determine the prognosis of cancer patients using H&E images is novel and suggests that the histology of tumors reflects their signaling activities. Since reports have shown that the signaling pathway activity of different cancers cannot be reflected simply by their mutational status^22–24^, the results of this study might encourage more research using imaging data to predict drug sensitivity according to targeted pathway activity.

This study employed transfer learning by adopting the pre-trained convolutional neural network ResNet101. However, the model could be further improved for accuracy and interpretability using the attention method in deep learning^25^. It is also noteworthy that because of the randomness of annotation in the training process, the trained models could be slightly different each time.

In conclusion, our study provides a novel machine learning model for cancer prognosis and theragnosis by predicting ER signaling activity in patients with ER + /HER2- breast cancer from whole-slide tumor H&E staining.

## Methods

### Gene expression data, H&E-stained images, and EERES

Level 3 gene expression profiles of 1082 breast cancer patients in TCGA Pan-Cancer were downloaded from cBioportal (https://cbioportal.org)^26,27^. The H&E-stained tumor images of 1077 (some of the patients included in gene expression data were not available for their tumor image) non-duplicated patients and their histological and pathological statuses were downloaded from the Genomic Data Commons Data Portal (https://portal.gdc.cancer.gov). The EERES for each sample were determined by GSVA with the gene set Hallmark Estrogen Response Early from MSigDB^13^. To elaborate, the gene expression matrix of all the samples and the list of the gene set Hallmark Estrogen Response Early from MSigDB (https://www.gsea-msigdb.org/gsea/msigdb/human/geneset/HALLMARK_ESTROGEN_RESPONSE_EARLY.html) with its corresponding 200 gene symbols were loaded into R package GSVA to determine the ER pathway activity score EERES. The gene expression matrix for all samples was generated from the gene expression level (TPM, transcript per million) of each gene in each sample using log2(TPM + 1) values.

### Data preparation

Out of the 1082 samples with gene expression profiles, 1077 samples had both gene expression profiles and whole-slide H&E-stained images. These 1077 samples were from four institutions. The dataset was split into training cohort (n = 265) using samples from three institutions—Harvard Medical School (Center code = 02), Lawrence Berkeley National Laboratory (Center code = 03), and Memorial Sloan-Kettering Cancer Center (Center code = 04). The trained model was then applied to the testing cohort (n = 812) from a single institution—Broad Institute of MIT (Massachusetts Institute of Technology) (Center code = 01). The downloaded images were cropped to square images using the length of the shorter side if their widths and lengths were not the same. The original images were then flipped horizontally or vertically according to their width or length and cropped to the same length as another square. The images were resized to 1024 × 1024 pixels. The images were labeled as 0 if the EERES was lower or equal to quantile 0.5 and 1 if it was higher than 0.5 (EERES threshold = -0.0376).

### Survival and statistical analyses

The Kaplan–Meier survival curves were plotted, and the log-rank statistics were determined using the Python package kaplanmeier. The optimal threshold for separating the survival time of the breast cancer patients was identified by searching for the most significant log-rank *p* value of PFS or OS durations using the predicted ER activity score in the range from quantile 0.1 to 0.9 (Table S4). Pearson statistics were determined using the Python library SciPy^28^. *p* value lower than 0.05 is considered significant. CoxPH method was implemented using Python package lifelines^29^ to generate a predicted risk score incorporating predicted ER activity score, age, and stage. A CoxPH model was fitted with data from the training cohort. The trained model was then used to predict the risk of the testing cohort using their predicted ER activity score, diagnostic age and stage. The most significant log-rank p-value was found by comparing the patients with predicted lower and higher risk of tumor progression using different quantile. The log-rank p-values between testing patients with low and high risk using different quantile are provided in Table S5.

### Model training and evaluation

The models were constructed and trained with the Python framework PyTorch^30^. A pre-trained ResNet101 with default parameters was employed^31^. The model was followed by fully connected layers with 128, 32, and 2 perceptrons, with a 0.2 dropout rate. The two perceptrons were followed by SoftMax for the probability of 0 and 1 classes. The loss was determined by CrossEntropyLoss, and the model was optimized by SGD (Stochastic Gradient Descent), with a 0.001 learning rate and a 0.9 momentum. The learning rate decay factor was 0.1 for every seven epochs. The training data were shuffled with random horizontal, vertical flip, and 360-degree rotation, with batch size 3. For each epoch, the model was evaluated with training data without augmentation.

The samples (n = 1077) were first separated into training (n = 812) and testing (n = 265) cohort first by their center code. A model was then trained with 30 epochs. The trained model was then used to predict the ER activity score for the testing cohort and determine the prognostic value of the predicted score.

### Ethical approval

This study used open-access TCGA data that were de-identified and accordance with informed consent documents by the NCBI and were thus exempted from IRB approval (https://www.cancer.gov/about-nci/organization/ccg/research/structural-genomics/tcga/history/policies/tcga-human-subjects-data-policies.pdf [accessed Mar-9–2023]).

### Supplementary Information

Supplementary Information 1.

Supplementary Tables.

## Data Availability

A html file of the Jupyter notebook used to generate the results is available in the supplementary information.

## Data availability

Processed data are available from the corresponding author upon reasonable request.
