# Supplementary material for: Deep learning-enabled breast cancer endocrine response determination from H&E staining based on ESR1 signaling activity
Source: Sci Rep. 2023 Dec 5;13:21454. doi: 10.1038/s41598-023-48830-x (PMC10698147; doi:10.1038/s41598-023-48830-x)
Supplement: Supplementary file 1 — Supplementary Information 1. [file 41598_2023_48830_MOESM1_ESM.html]

Code


# Prognosis prediction based on estrogen receptor signaling activity from H&E staining by deep learning¶

Chun Wai Ng, Kwong-kwok Wong

In [2]:

```
import sys
print("Python version:", sys.version)
!pip list | grep 'slideio\|pandas\|numpy\|matplotlib\|rpy2\|torch\|torchvision\|sklearn\|scipy\|kaplanmeier\|lifelines\|rpy2'
import slideio
import pandas as pd
import numpy as np
import matplotlib.pyplot as plt
import matplotlib.image
import os
import math
import rpy2
import json
import torch
torch.manual_seed(0)
from torch.utils.data import DataLoader, Dataset
import torch.nn as nn
import torch.optim as optim
from torch.optim import lr_scheduler
import torch.backends.cudnn as cudnn
import torchvision
from torchvision.models import ResNet50_Weights
from torchvision import datasets, models, transforms
import torchvision.transforms.functional as F
from PIL import Image
import matplotlib.pyplot as plt
import time
import os
import copy
from sklearn.model_selection import train_test_split
import scipy
from sklearn.model_selection import KFold
from sklearn.metrics import roc_auc_score, roc_curve, RocCurveDisplay
from scipy.stats import spearmanr, pearsonr
from lifelines import CoxPHFitter
import kaplanmeier as km
import rpy2.robjects as ro
from rpy2.robjects.packages import importr
from rpy2.robjects import pandas2ri
import rpy2.robjects as robjects
from rpy2.robjects.conversion import localconverter
rinstalled = robjects.globalenv.find("installed.packages")
rversion = robjects.globalenv.find("R.Version")
rpkgs = rinstalled()
rvers = rpkgs.rx(robjects.StrVector(["GSVA"]), robjects.StrVector(["Version"]))
print(rversion().rx2("version.string"))
print("GSVA Version:", rvers)

def gsva(geneExpressionProfile, gene_sets):
    rbase = importr('base')
    print("Converting df")
    with localconverter(ro.default_converter + pandas2ri.converter):
        geneExpressionProfile_r = ro.conversion.py2rpy(geneExpressionProfile)
    gene_sets_r = ro.ListVector(gene_sets)
    gsvar = importr("GSVA")
    es = gsvar.gsva(rbase.as_matrix(geneExpressionProfile_r), gene_sets_r)
    es_df = pd.DataFrame(np.array(es.transpose()), index=es.colnames, columns=es.rownames)
    return es_df

def finding_best_threshold_with_FS_SS(fs_eeres_df, ss_eeres_df, survival_type, value_name, q01, q09, stratify_name):
    bestp = 1
    bestq = 0
    qs = []
    for q in np.arange(q01, q09, 0.01):
        result1 = km.fit(fs_eeres_df[f'{survival_type[0]}_MONTHS'], fs_eeres_df[f'{survival_type[0]}_STATUS'], (fs_eeres_df[value_name]>q).apply(lambda x: "Higher EERES" if x else "Lower EERES"))
        result2 = km.fit(ss_eeres_df[f'{survival_type[1]}_MONTHS'], ss_eeres_df[f'{survival_type[1]}_STATUS'], (ss_eeres_df[value_name]>q).apply(lambda x: "Hihger EERES" if x else "Lower EERES"))
        average_p = (result1["logrank_P"]+result2["logrank_P"])/2
        if (result1["logrank_P"]<0.05 or result2["logrank_P"]<0.05):
            qs.append(q)
        if (result1["logrank_P"]<0.05 or result2["logrank_P"]<0.05) and average_p < bestp:
            bestp=average_p
            bestq=q
    if bestp==1:
        print('not significant')
    else:
        print(f"Best {value_name} threshold:", bestq)
        result = km.fit(fs_eeres_df[f'{survival_type[0]}_MONTHS'], fs_eeres_df[f'{survival_type[0]}_STATUS'], (fs_eeres_df[value_name]>bestq).apply(lambda x: f"Higher {stratify_name}" if x else f"Lower {stratify_name}"))
        km.plot(result, title=f"{survival_type[0]} of ER+/HER2- stratified by {stratify_name}, Logrank p-value={result['logrank_P']:.3e}", full_ylim=True, y_percentage=True)
        plt.show()
        result = km.fit(ss_eeres_df[f'{survival_type[1]}_MONTHS'], ss_eeres_df[f'{survival_type[1]}_STATUS'], (ss_eeres_df[value_name]>bestq).apply(lambda x: f"Higher {stratify_name}" if x else f"Lower {stratify_name}"))
        km.plot(result, title=f"{survival_type[1]} of ER+/HER2- stratified by {stratify_name}, Logrank p-value={result['logrank_P']:.3e}", full_ylim=True, y_percentage=True)
        plt.show()
#         for q in qs:
#             print(q)
#             result = km.fit(fs_eeres_df[f'{survival_type[0]}_MONTHS'], fs_eeres_df[f'{survival_type[0]}_STATUS'], (fs_eeres_df[value_name]>q).apply(lambda x: f"Higher {stratify_name}" if x else f"Lower {stratify_name}"))
#             km.plot(result, title=f"{survival_type[0]} of ER+/HER2- stratified by {stratify_name}, Logrank p-value={result['logrank_P']:.3e}", full_ylim=True, y_percentage=True)
#             plt.show()
#             result = km.fit(ss_eeres_df[f'{survival_type[1]}_MONTHS'], ss_eeres_df[f'{survival_type[1]}_STATUS'], (ss_eeres_df[value_name]>q).apply(lambda x: f"Higher {stratify_name}" if x else f"Lower {stratify_name}"))
#             km.plot(result, title=f"{survival_type[1]} of ER+/HER2- stratified by {stratify_name}, Logrank p-value={result['logrank_P']:.3e}", full_ylim=True, y_percentage=True)
#             plt.show()
```

```
Python version: 3.10.12 (main, Jun 11 2023, 05:26:28) [GCC 11.4.0]
kaplanmeier              0.1.9
lifelines                0.27.7
matplotlib               3.5.1
matplotlib-inline        0.1.3
numpy                    1.23.0
numpyro                  0.13.2
pandas                   2.0.3
pytorch-lightning        2.1.0
rpy2                     3.5.5
scipy                    1.11.2
slideio                  2.2.0
torch                    2.0.1
torchmetrics             1.2.0
torchvision              0.15.2
[1] "R version 4.3.1 (2023-06-16)"

GSVA Version: [1] "1.48.3"
```

## Image resizing, augmentating and cropping¶

## Loading file metadata and file locations¶

In [3]:

```
meta_center = pd.read_table('../gdc_manifest_20231018_160851.txt', encoding='utf-8')
meta_center['Center'] = [f[17:19] for f in meta_center['filename']]
meta_center['Case ID'] = [f[:12] for f in meta_center['filename']]
meta_center = meta_center.set_index('Case ID')
meta_center = meta_center[~meta_center.index.duplicated(keep='first')]
prefix = '../Images/'
meta_nondup = meta_center
meta_nondup = meta_nondup.rename(columns={'id':'File ID','filename':'File Name'})
# meta_nondup.to_csv("Table S2_new.csv")
```

In [4]:

```
# prefix = '../../../Untitled/BRCA-EERES imaging/Analysis/Images/'
# meta_dx = pd.read_table('../../../Untitled/BRCA-EERES imaging/Analysis/File_metadata.tsv', encoding='utf-8')
# meta_dx['Case ID'] = [f[:12] for f in meta_dx['File Name']]
# meta_nondup = meta_dx[~meta_dx['Case ID'].duplicated(keep='first')]
# meta_nondup = meta_nondup.set_index('Case ID') # Get images from unique patients
# # files = ("../../../Untitled/BRCA-EERES imaging/Analysis/Images" + meta_nondup["File ID"] + '/' + meta_nondup["File Name"] + "-1.jpg").values
# meta_nondup.to_csv("Table S2_new.csv")
# # meta_nondup = pd.read_csv('../Figures, Tables and Supplemental Data/Table S2.csv',index_col=0,skiprows=1)
# meta_nondup = meta_nondup.join(meta_center[['Center']],how='inner')
# meta_nondup
```

org\_files = ("../../../Untitled/BRCA-EERES imaging/Analysis/Images/" + meta\_nondup["File ID"] + '/' + meta\_nondup["File Name"]).values # Get the image file locations
for i, file in enumerate(org\_files):
print(i,file)
slide = slideio.open\_slide(file,'SVS')
print("slide")
num\_scenes = slide.num\_scenes
print("num")
scene = slide.get\_scene(0)
print("scene")
rect = scene.rect
print(rect)
size=512
image = scene.read\_block(size=(int(size*rect[2]/rect[3]) if rect[2]>rect[3] else size, int(size*rect[3]/rect[2]) if rect[3]>rect[2] else size))
image\_tr = scipy.ndimage.rotate(image, 180)
print("image")
block1 = image[0:size, 0:size, 0:3]
block2 = image\_tr[0:size, 0:size, 0:3]
matplotlib.image.imsave(file+"\_512-1.jpg", block1)
matplotlib.image.imsave(file+"\_512-2.jpg", block2)
matplotlib.image.imsave(file+"\_512.jpg", image)
print("save")

## Loading TCGA Pan-Cancer BRCA level 3 gene expression data from cBioPortal¶

In [5]:

```
brca_lv3 = pd.read_csv("../brca_tcga_pan_can_atlas_2018/data_mrna_seq_v2_rsem.txt", index_col=0, sep='\t').groupby(level=0).mean().transpose().iloc[1:]
brca_lv3.index = [i[:12] for i in brca_lv3.index]
brca_lv3
```

Out[5]:

| Hugo\_Symbol | A1BG | A1CF | A2BP1 | A2LD1 | A2M | A2M-AS1 | A2ML1 | A4GALT | A4GNT | AAA1 | ... | ZWINT | ZXDA | ZXDB | ZXDC | ZYG11A | ZYG11B | ZYX | ZZEF1 | ZZZ3 | psiTPTE22 |
| --- | --- | --- | --- | --- | --- | --- | --- | --- | --- | --- | --- | --- | --- | --- | --- | --- | --- | --- | --- | --- | --- |
| TCGA-3C-AAAU | 197.090 | 0.0000 | 0.0000 | 102.9630 | 5798.37 | 32.2187 | 1.3786 | 68.2424 | 8.6165 | 0.3447 | ... | 931.957 | 129.5920 | 1007.780 | 1658.500 | 258.4940 | 1208.370 | 3507.25 | 1894.930 | 1180.460 | 1.7233 |
| TCGA-3C-AALI | 237.384 | 0.0000 | 0.0000 | 70.8646 | 7571.98 | 29.9782 | 4.3502 | 157.6940 | 0.5438 | 0.0000 | ... | 965.198 | 59.8151 | 448.613 | 1343.120 | 198.4770 | 603.589 | 5504.62 | 1318.650 | 406.743 | 926.5910 |
| TCGA-3C-AALJ | 423.237 | 0.9066 | 0.0000 | 161.2600 | 8840.40 | 17.2620 | 0.0000 | 573.8890 | 0.0000 | 0.0000 | ... | 2531.280 | 35.3581 | 533.998 | 768.812 | 331.8220 | 532.185 | 5458.75 | 942.883 | 509.519 | 35.3581 |
| TCGA-3C-AALK | 191.018 | 0.0000 | 0.0000 | 62.5072 | 10960.20 | 17.8527 | 1.6549 | 506.4130 | 0.0000 | 0.0000 | ... | 668.597 | 55.0269 | 437.733 | 863.881 | 175.4240 | 607.365 | 5691.35 | 781.134 | 700.869 | 66.6115 |
| TCGA-4H-AAAK | 268.881 | 0.4255 | 3.8298 | 154.3700 | 9585.44 | 31.5787 | 3.4043 | 342.1280 | 0.4255 | 0.4255 | ... | 674.468 | 48.9362 | 424.255 | 1049.790 | 14.0426 | 775.745 | 4041.70 | 831.915 | 881.702 | 187.2340 |
| ... | ... | ... | ... | ... | ... | ... | ... | ... | ... | ... | ... | ... | ... | ... | ... | ... | ... | ... | ... | ... | ... |
| TCGA-WT-AB44 | 471.285 | 0.0000 | 0.0000 | 61.7308 | 5409.31 | 39.6823 | 6.5160 | 356.7500 | 0.8145 | 0.0000 | ... | 180.819 | 24.4349 | 243.535 | 772.959 | 98.5543 | 315.211 | 10937.10 | 800.652 | 443.087 | 724.9030 |
| TCGA-XX-A899 | 223.220 | 0.0000 | 0.3937 | 131.2280 | 20348.80 | 27.2283 | 0.3937 | 505.5120 | 0.7874 | 0.0000 | ... | 457.087 | 70.8661 | 643.701 | 1266.540 | 21.2598 | 688.189 | 5118.11 | 1933.860 | 670.079 | 98.4252 |
| TCGA-XX-A89A | 255.135 | 2.3618 | 1.4171 | 79.9291 | 17094.80 | 31.7572 | 55.7393 | 615.4940 | 2.8342 | 0.9447 | ... | 694.379 | 48.6538 | 341.521 | 1375.530 | 164.3840 | 746.812 | 5477.56 | 1437.410 | 953.708 | 235.2380 |
| TCGA-Z7-A8R5 | 439.543 | 0.0000 | 0.5973 | 81.3010 | 36838.50 | 84.0964 | 2.3893 | 456.3510 | 0.0000 | 0.0000 | ... | 258.639 | 32.2552 | 248.484 | 796.225 | 51.9667 | 505.928 | 6675.63 | 754.413 | 750.829 | 238.9270 |
| TCGA-Z7-A8R6 | 248.327 | 0.0000 | 0.0000 | 25.1866 | 7339.17 | 8.3723 | 4.1757 | 768.6820 | 0.0000 | 0.0000 | ... | 2435.840 | 34.4498 | 389.735 | 947.890 | 139.5390 | 573.467 | 3402.52 | 564.419 | 462.114 | 20.8786 |

1082 rows × 20511 columns

## Early Estrogen Response Enrichment Scores (EERES) by GSVA¶

In [6]:

```
# h_gs = json.loads(open("h.all.v2023.1.Hs.json", 'r').read())
# early_es_gs = {'HALLMARK_ESTROGEN_RESPONSE_EARLY': h_gs['HALLMARK_ESTROGEN_RESPONSE_EARLY']['geneSymbols']}
# brca_earlyes_es = gsva((brca_lv3+1).applymap(math.log2).transpose(), early_es_gs)
# brca_earlyes_es.index = [i[:12] for i in brca_earlyes_es.index]
# brca_earlyes_es.to_csv("Table S1.csv")
brca_earlyes_es = pd.read_csv("Table S1.csv", index_col=0)
meta_nondup = meta_nondup.join(brca_earlyes_es,how='inner')
meta_nondup.to_csv("Table S2.csv")
meta_nondup
```

Out[6]:

|  | File ID | File Name | md5 | size | state | Center | HALLMARK\_ESTROGEN\_RESPONSE\_EARLY |
| --- | --- | --- | --- | --- | --- | --- | --- |
| TCGA-BH-A18N | 2ed1ad16-98b3-4941-b223-47a1af8efdae | TCGA-BH-A18N-11A-02-TSB.c1360bc0-7e02-4847-a9d... | 6d52ff8616741c806b5b69a8a8aa2468 | 68191817 | validated | 02 | 0.289838 |
| TCGA-GM-A2DD | 2f01db43-018f-4c39-b081-36ef73ade5a0 | TCGA-GM-A2DD-01A-01-TSA.A6FD944E-FDD8-4D2F-830... | f54444592c5149ae78dab143552d95b6 | 139355039 | validated | 01 | -0.237541 |
| TCGA-C8-A12Z | 2f7f3248-44d7-49da-b63a-8824f4bf6e81 | TCGA-C8-A12Z-01A-01-TSA.d85a700f-8886-4b59-8ce... | b996def520ee1dbb447fc1439f021884 | 214185277 | validated | 01 | -0.025701 |
| TCGA-AC-A2FK | 2f7a479b-185f-4948-b886-ff702ad7f84b | TCGA-AC-A2FK-01A-01-TS1.807C5D36-7171-495C-B6A... | 0c9193deb55553e8beed31d0b06c6e84 | 402847361 | validated | 01 | 0.000622 |
| TCGA-E9-A1NF | 2fc8b8d8-f721-495a-9d6e-574aab448b2a | TCGA-E9-A1NF-01A-01-TSA.c5078c11-f3d8-47d5-b02... | a7904f0270c5071f937f7e91be1480c8 | 335548571 | validated | 01 | -0.134763 |
| ... | ... | ... | ... | ... | ... | ... | ... |
| TCGA-D8-A27M | 06efedbc-f917-4ffa-a9ed-804f167dbe5b | TCGA-D8-A27M-01A-01-TSA.67f12b28-f756-49c8-a7f... | f915734eeaca979c9ae90f0607aa33f4 | 97085691 | validated | 01 | -0.283343 |
| TCGA-D8-A27P | 07a95a70-bea1-4ab8-9fbc-01b00b79b351 | TCGA-D8-A27P-01A-01-TSA.df327c6c-1fde-4c29-ad3... | dc6b9e0c70abbe809a52ac856c16559c | 95657621 | validated | 01 | 0.210204 |
| TCGA-EW-A1IY | 0839c5d2-3d96-49f8-bfd6-7b3d05188365 | TCGA-EW-A1IY-01A-01-TSA.2f6f198d-844c-442d-956... | 45d45497d752312fe5167fbb827bad08 | 271760687 | validated | 01 | -0.202351 |
| TCGA-OL-A5RZ | 0909b73e-1346-48f6-8279-a8add8993965 | TCGA-OL-A5RZ-01A-01-TSA.58A5566D-6342-4BCB-B22... | c9d15d65952f77de84c0afdb49da604a | 160114423 | validated | 01 | -0.318510 |
| TCGA-OL-A6VQ | 09d2afd1-25d2-4d5b-9044-d16cbf14304d | TCGA-OL-A6VQ-01A-01-TSA.0CD11145-841A-4FE3-BAC... | abb76704ccd8234aae87b93eb8310abd | 197220271 | validated | 01 | 0.544215 |

1077 rows × 7 columns

## Loading TCGA BRCA IHC data from GDC Portal¶

In [7]:

```
meta_erihc = pd.read_table("nationwidechildrens.org_clinical_patient_brca.txt", sep='\t', index_col=1).iloc[2:]
meta_erihc

# Joining IHC data with ESR1/EERES data
eeres_ihc_df = brca_earlyes_es.join(meta_erihc, how='inner')
eeres_ihc_df["Subtype"] = None
eeres_ihc_df["Subtype"][(eeres_ihc_df["er_status_by_ihc"]=='Positive') & (eeres_ihc_df["her2_status_by_ihc"]=='Negative')] = "ER+/HER2-"
eeres_ihc_df["Subtype"][(eeres_ihc_df["er_status_by_ihc"]=='Negative') & (eeres_ihc_df["her2_status_by_ihc"]=='Negative') & (eeres_ihc_df["pr_status_by_ihc"]=='Negative')] = "TNBC"
eeres_ihc_df_erposerbb2neg = eeres_ihc_df[eeres_ihc_df["Subtype"]=="ER+/HER2-"]
eeres_ihc_df_erposerbb2neg
eeres_ihc_df_tnbc = eeres_ihc_df[eeres_ihc_df["Subtype"]=="TNBC"]
eeres_ihc_df["EERES"] = brca_earlyes_es.loc[eeres_ihc_df.index]
eeres_ihc_df["ESR1"] = brca_lv3.loc[eeres_ihc_df.index]["ESR1"]
eeres_ihc_df
```

```
/tmp/ipykernel_54612/1856840598.py:7: SettingWithCopyWarning: 
A value is trying to be set on a copy of a slice from a DataFrame

See the caveats in the documentation: https://pandas.pydata.org/pandas-docs/stable/user_guide/indexing.html#returning-a-view-versus-a-copy
  eeres_ihc_df["Subtype"][(eeres_ihc_df["er_status_by_ihc"]=='Positive') & (eeres_ihc_df["her2_status_by_ihc"]=='Negative')] = "ER+/HER2-"
/tmp/ipykernel_54612/1856840598.py:8: SettingWithCopyWarning: 
A value is trying to be set on a copy of a slice from a DataFrame

See the caveats in the documentation: https://pandas.pydata.org/pandas-docs/stable/user_guide/indexing.html#returning-a-view-versus-a-copy
  eeres_ihc_df["Subtype"][(eeres_ihc_df["er_status_by_ihc"]=='Negative') & (eeres_ihc_df["her2_status_by_ihc"]=='Negative') & (eeres_ihc_df["pr_status_by_ihc"]=='Negative')] = "TNBC"
```

Out[7]:

|  | HALLMARK\_ESTROGEN\_RESPONSE\_EARLY | bcr\_patient\_uuid | form\_completion\_date | prospective\_collection | retrospective\_collection | birth\_days\_to | gender | menopause\_status | race | ethnicity | ... | metastatic\_tumor\_indicator | patient\_id | project\_code | site\_of\_primary\_tumor\_other | stage\_other | tissue\_source\_site | tumor\_tissue\_site | Subtype | EERES | ESR1 |
| --- | --- | --- | --- | --- | --- | --- | --- | --- | --- | --- | --- | --- | --- | --- | --- | --- | --- | --- | --- | --- | --- |
| TCGA-3C-AAAU | 0.001404 | 6E7D5EC6-A469-467C-B748-237353C23416 | 2014-1-13 | NO | YES | -20211 | FEMALE | Pre (<6 months since LMP AND no prior bilatera... | WHITE | NOT HISPANIC OR LATINO | ... | [Not Available] | AAAU | [Not Available] | [Not Applicable] | [Not Available] | 3C | Breast | ER+/HER2- | 0.001404 | 3457.9600 |
| TCGA-3C-AALI | -0.409362 | 55262FCB-1B01-4480-B322-36570430C917 | 2014-7-28 | NO | YES | -18538 | FEMALE | Post (prior bilateral ovariectomy OR >12 mo si... | BLACK OR AFRICAN AMERICAN | NOT HISPANIC OR LATINO | ... | [Not Available] | AALI | [Not Available] | [Not Applicable] | [Not Available] | 3C | Breast | None | -0.409362 | 68.5155 |
| TCGA-3C-AALJ | -0.038938 | 427D0648-3F77-4FFC-B52C-89855426D647 | 2014-7-28 | NO | YES | -22848 | FEMALE | Post (prior bilateral ovariectomy OR >12 mo si... | BLACK OR AFRICAN AMERICAN | NOT HISPANIC OR LATINO | ... | [Not Available] | AALJ | [Not Available] | [Not Applicable] | [Not Available] | 3C | Breast | None | -0.038938 | 7482.3200 |
| TCGA-3C-AALK | 0.356928 | C31900A4-5DCD-4022-97AC-638E86E889E4 | 2014-7-28 | NO | YES | -19074 | FEMALE | [Unknown] | BLACK OR AFRICAN AMERICAN | NOT HISPANIC OR LATINO | ... | [Not Available] | AALK | [Not Available] | [Not Applicable] | [Not Available] | 3C | Breast | None | 0.356928 | 2485.3100 |
| TCGA-4H-AAAK | 0.154434 | 6623FC5E-00BE-4476-967A-CBD55F676EA6 | 2014-11-13 | YES | NO | -18371 | FEMALE | Post (prior bilateral ovariectomy OR >12 mo si... | WHITE | NOT HISPANIC OR LATINO | ... | [Not Available] | AAAK | [Not Available] | [Not Applicable] | [Not Available] | 4H | Breast | None | 0.154434 | 5518.3000 |
| ... | ... | ... | ... | ... | ... | ... | ... | ... | ... | ... | ... | ... | ... | ... | ... | ... | ... | ... | ... | ... | ... |
| TCGA-WT-AB44 | -0.224513 | 5CD79093-1571-4F71-8136-0D84CCABDCAC | 2014-7-16 | NO | YES | [Not Available] | FEMALE | Post (prior bilateral ovariectomy OR >12 mo si... | WHITE | NOT HISPANIC OR LATINO | ... | [Not Available] | AB44 | [Not Available] | [Not Applicable] | [Not Available] | WT | Breast | ER+/HER2- | -0.224513 | 4558.7500 |
| TCGA-XX-A899 | 0.161219 | F89588E9-CA73-4465-A7FB-7246EDB45E3A | 2014-2-21 | NO | YES | -17022 | FEMALE | Post (prior bilateral ovariectomy OR >12 mo si... | WHITE | NOT HISPANIC OR LATINO | ... | [Not Available] | A899 | [Not Available] | [Not Applicable] | [Not Available] | XX | Breast | ER+/HER2- | 0.161219 | 2731.5000 |
| TCGA-XX-A89A | -0.122835 | CA20249F-B7EA-4FD9-9ECB-34F74755AE35 | 2014-2-21 | NO | YES | -25000 | FEMALE | Post (prior bilateral ovariectomy OR >12 mo si... | WHITE | NOT HISPANIC OR LATINO | ... | [Not Available] | A89A | [Not Available] | [Not Applicable] | [Not Available] | XX | Breast | ER+/HER2- | -0.122835 | 2499.7600 |
| TCGA-Z7-A8R5 | -0.193181 | 23F438BD-1DBB-4D46-972F-1E8E74DDBD37 | 2014-7-9 | NO | YES | -22280 | FEMALE | Post (prior bilateral ovariectomy OR >12 mo si... | WHITE | NOT HISPANIC OR LATINO | ... | [Not Available] | A8R5 | [Not Available] | [Not Applicable] | [Not Available] | Z7 | Breast | ER+/HER2- | -0.193181 | 3301.3800 |
| TCGA-Z7-A8R6 | 0.209939 | B1D44C81-747D-471F-9093-AEB262A17975 | 2014-7-9 | NO | YES | -16955 | FEMALE | Pre (<6 months since LMP AND no prior bilatera... | WHITE | NOT HISPANIC OR LATINO | ... | [Not Available] | A8R6 | [Not Available] | [Not Applicable] | [Not Available] | Z7 | Breast | ER+/HER2- | 0.209939 | 6429.9300 |

1081 rows × 115 columns

In [8]:

```
# Change clinical stages to int

np.unique(eeres_ihc_df['ajcc_pathologic_tumor_stage'])

def stage_to_int(s):
    match s:
        case 'Stage I':
            return 1
        case 'Stage IA':
            return 1
        case 'Stage IB':
            return 1
        case 'Stage II':
            return 2
        case 'Stage IIA':
            return 2
        case 'Stage IIB':
            return 2
        case 'Stage III':
            return 3
        case 'Stage IIIA':
            return 3
        case 'Stage IIIB':
            return 3
        case 'Stage IIIC':
            return 3
        case 'Stage IV':
            return 4
        case 'Stage X':
            return 0
        case '[Discrepancy]':
            return 0
        case '[Not Available]':
            return 0

meta_erihc['ajcc_pathologic_tumor_stage'] = meta_erihc['ajcc_pathologic_tumor_stage'].apply(stage_to_int)
```

## Loading survival data from cBioPortal¶

In [9]:

```
brca_clinical = pd.read_table("../brca_tcga_pan_can_atlas_2018/data_clinical_patient.txt", index_col=0, skiprows=4)
brca_clinical = brca_clinical[["OS_STATUS", "OS_MONTHS","PFS_STATUS", "PFS_MONTHS", "DFS_MONTHS", "DFS_STATUS", "DSS_MONTHS", "DSS_STATUS"]]
brca_clinical["OS_STATUS"] = [s[0] for s in brca_clinical["OS_STATUS"]]
brca_clinical["PFS_STATUS"] = [str(s)[0] for s in brca_clinical["PFS_STATUS"]]
brca_clinical["DFS_STATUS"] = [str(s)[0] for s in brca_clinical["DFS_STATUS"]]
brca_clinical["DSS_STATUS"] = [s[0] if str(s) != 'nan' else np.nan for s in brca_clinical["DSS_STATUS"]]
```

In [10]:

```
brca_clinical
```

Out[10]:

|  | OS\_STATUS | OS\_MONTHS | PFS\_STATUS | PFS\_MONTHS | DFS\_MONTHS | DFS\_STATUS | DSS\_MONTHS | DSS\_STATUS |
| --- | --- | --- | --- | --- | --- | --- | --- | --- |
| PATIENT\_ID |  |  |  |  |  |  |  |  |
| TCGA-3C-AAAU | 0 | 133.050597 | 1 | 59.440444 | 59.440444 | 1 | 133.050597 | 0 |
| TCGA-3C-AALI | 0 | 131.669790 | 0 | 131.669790 | 131.669790 | 0 | 131.669790 | 0 |
| TCGA-3C-AALJ | 0 | 48.459743 | 0 | 48.459743 | 48.459743 | 0 | 48.459743 | 0 |
| TCGA-3C-AALK | 0 | 47.604958 | 0 | 47.604958 | NaN | n | 47.604958 | 0 |
| TCGA-4H-AAAK | 0 | 11.440971 | 0 | 11.440971 | 11.440971 | 0 | 11.440971 | 0 |
| ... | ... | ... | ... | ... | ... | ... | ... | ... |
| TCGA-WT-AB44 | 0 | 29.029819 | 0 | 29.029819 | 29.029819 | 0 | 29.029819 | 0 |
| TCGA-XX-A899 | 0 | 15.353256 | 0 | 15.353256 | 15.353256 | 0 | 15.353256 | 0 |
| TCGA-XX-A89A | 0 | 16.043660 | 0 | 16.043660 | 16.043660 | 0 | 16.043660 | 0 |
| TCGA-Z7-A8R5 | 0 | 108.064569 | 1 | 5.950620 | NaN | n | 108.064569 | 0 |
| TCGA-Z7-A8R6 | 0 | 107.045402 | 0 | 107.045402 | 107.045402 | 0 | 107.045402 | 0 |

1084 rows × 8 columns

## Joining survival data with EERES\_IHC data¶

### Show the survival of ER+/HER2- and TNBC of all data¶

In [11]:

```
brca_clinical_eeres_ihc_df = brca_clinical.join(eeres_ihc_df, how='inner')

brca_clinical_dfs = brca_clinical_eeres_ihc_df[["DFS_MONTHS", "DFS_STATUS", "HALLMARK_ESTROGEN_RESPONSE_EARLY", "Subtype", "EERES", "ESR1"]].dropna()
brca_clinical_dss = brca_clinical_eeres_ihc_df[["DSS_MONTHS", "DSS_STATUS", "HALLMARK_ESTROGEN_RESPONSE_EARLY", "Subtype", "EERES", "ESR1"]].dropna()
brca_clinical_pfs = brca_clinical_eeres_ihc_df[["PFS_MONTHS", "PFS_STATUS", "HALLMARK_ESTROGEN_RESPONSE_EARLY", "Subtype", "EERES", "ESR1"]].dropna()
brca_clinical_os = brca_clinical_eeres_ihc_df[["OS_MONTHS", "OS_STATUS", "HALLMARK_ESTROGEN_RESPONSE_EARLY", "Subtype", "EERES", "ESR1"]].dropna()


result = km.fit(brca_clinical_dfs['DFS_MONTHS'], brca_clinical_dfs['DFS_STATUS'], brca_clinical_dfs["Subtype"])
km.plot(result, title=f"DFS of ER+/HER2- vs TNBC, Logrank p-value={result['logrank_P']:.3e}", full_ylim=True, y_percentage=True)
plt.show()
result = km.fit(brca_clinical_dss['DSS_MONTHS'], brca_clinical_dss['DSS_STATUS'], brca_clinical_dss["Subtype"])
km.plot(result, title=f"DSS of ER+/HER2- vs TNBC, Logrank p-value={result['logrank_P']:.3e}", full_ylim=True, y_percentage=True)
plt.show()
result = km.fit(brca_clinical_pfs['PFS_MONTHS'], brca_clinical_pfs['PFS_STATUS'], brca_clinical_pfs["Subtype"])
km.plot(result, title=f"PFS of ER+/HER2- vs TNBC, Logrank p-value={result['logrank_P']:.3e}", full_ylim=True, y_percentage=True)
plt.show()
result = km.fit(brca_clinical_os['OS_MONTHS'], brca_clinical_os['OS_STATUS'], brca_clinical_os["Subtype"])
km.plot(result, title=f"OS of ER+/HER2- vs TNBC, Logrank p-value={result['logrank_P']:.3e}", full_ylim=True, y_percentage=True)
plt.show()
plt.scatter(eeres_ihc_df[eeres_ihc_df["Subtype"]=="ER+/HER2-"]["ESR1"].apply(math.log), eeres_ihc_df[eeres_ihc_df["Subtype"]=="ER+/HER2-"]["EERES"])
# plt.scatter(eeres_ihc_df[eeres_ihc_df["Subtype"]=="ERBB2+"]["esr1"].apply(math.log), eeres_ihc_df[eeres_ihc_df["Subtype"]=="ERBB2+"]["eeres"])
plt.scatter(eeres_ihc_df[eeres_ihc_df["Subtype"]=="TNBC"]["ESR1"].apply(math.log), eeres_ihc_df[eeres_ihc_df["Subtype"]=="TNBC"]["EERES"])


plt.legend(["ER+/HER2-", "TNBC"])
plt.xlabel("ESR1")
plt.ylabel("EERES")
plt.show()
```

## Getting the best thershold of EERES for ER+/HER2- and TNBC patients¶

In [12]:

```
brca_clinical_erposerbb2neg = brca_clinical_eeres_ihc_df[brca_clinical_eeres_ihc_df['Subtype']=='ER+/HER2-']

brca_clinical_erposerbb2neg_dfs = brca_clinical_erposerbb2neg[["DFS_MONTHS", "DFS_STATUS", "HALLMARK_ESTROGEN_RESPONSE_EARLY"]].dropna()
brca_clinical_erposerbb2neg_dss = brca_clinical_erposerbb2neg[["DSS_MONTHS", "DSS_STATUS", "HALLMARK_ESTROGEN_RESPONSE_EARLY"]].dropna()
brca_clinical_erposerbb2neg_pfs = brca_clinical_erposerbb2neg[["PFS_MONTHS", "PFS_STATUS", "HALLMARK_ESTROGEN_RESPONSE_EARLY"]].dropna()
brca_clinical_erposerbb2neg_os = brca_clinical_erposerbb2neg[["OS_MONTHS", "OS_STATUS", 'HALLMARK_ESTROGEN_RESPONSE_EARLY']].dropna()
```

### ER+/HER2-¶

In [13]:

```
print("DFS/DSS")
finding_best_threshold_with_FS_SS(brca_clinical_erposerbb2neg_dfs, brca_clinical_erposerbb2neg_dss, ["DFS", "DSS"], 'HALLMARK_ESTROGEN_RESPONSE_EARLY', brca_earlyes_es["HALLMARK_ESTROGEN_RESPONSE_EARLY"].quantile(0.1), brca_earlyes_es["HALLMARK_ESTROGEN_RESPONSE_EARLY"].quantile(0.9), "EERES")

# Figure 2
print("PFS/OS")
finding_best_threshold_with_FS_SS(brca_clinical_erposerbb2neg_pfs, brca_clinical_erposerbb2neg_os, ["PFS", "OS"], 'HALLMARK_ESTROGEN_RESPONSE_EARLY', brca_earlyes_es["HALLMARK_ESTROGEN_RESPONSE_EARLY"].quantile(0.1), brca_earlyes_es["HALLMARK_ESTROGEN_RESPONSE_EARLY"].quantile(0.9), "EERES")
```

```
DFS/DSS
Best HALLMARK_ESTROGEN_RESPONSE_EARLY threshold: 0.2020931077758909
```

```
PFS/OS
Best HALLMARK_ESTROGEN_RESPONSE_EARLY threshold: 0.2020931077758909
```

### TNBC¶

In [14]:

```
brca_clinical_tnbc = brca_clinical_eeres_ihc_df[brca_clinical_eeres_ihc_df['Subtype']=='TNBC']

brca_clinical_tnbc_dfs = brca_clinical_tnbc[["DFS_MONTHS", "DFS_STATUS", "HALLMARK_ESTROGEN_RESPONSE_EARLY"]].dropna()
brca_clinical_tnbc_dss = brca_clinical_tnbc[["DSS_MONTHS", "DSS_STATUS", "HALLMARK_ESTROGEN_RESPONSE_EARLY"]].dropna()
brca_clinical_tnbc_pfs = brca_clinical_tnbc[["PFS_MONTHS", "PFS_STATUS", "HALLMARK_ESTROGEN_RESPONSE_EARLY"]].dropna()
brca_clinical_tnbc_os = brca_clinical_tnbc[["OS_MONTHS", "OS_STATUS", 'HALLMARK_ESTROGEN_RESPONSE_EARLY']].dropna()

print("DFS/DSS")
finding_best_threshold_with_FS_SS(brca_clinical_tnbc_dfs, brca_clinical_tnbc_dss, ["DFS", "DSS"], 'HALLMARK_ESTROGEN_RESPONSE_EARLY', brca_earlyes_es["HALLMARK_ESTROGEN_RESPONSE_EARLY"].quantile(0.1), brca_earlyes_es["HALLMARK_ESTROGEN_RESPONSE_EARLY"].quantile(0.9), "EERES")
print("PFS/OS")
finding_best_threshold_with_FS_SS(brca_clinical_tnbc_pfs, brca_clinical_tnbc_os, ["PFS", "OS"], 'HALLMARK_ESTROGEN_RESPONSE_EARLY', brca_earlyes_es["HALLMARK_ESTROGEN_RESPONSE_EARLY"].quantile(0.1), brca_earlyes_es["HALLMARK_ESTROGEN_RESPONSE_EARLY"].quantile(0.9), "EERES")
```

```
DFS/DSS
not significant
PFS/OS
not significant
```

## Preparing trainig and testing data¶

In [15]:

```
# Data augmentation and normalization for training
device = torch.device("cuda" if torch.cuda.is_available() else "cpu")
class MyData_train(Dataset):
    def __init__(self, X, y):
        self.X = []
        for x in X:
            self.X.append(torch.tensor(np.moveaxis(np.array(Image.open(x)), -1, 0).astype(float)).to(device))
        self.classes = np.unique(y)
        self.y = np.array(y)
        self.tumorid = y.index

    def __len__(self):
        return len(self.X)

    def __getitem__(self, index):
        transform = transforms.Compose([
#                                         transforms.RandomAffine(degrees=(0,360),translate=(0.1,0.3)),
                                        transforms.RandomRotation(360),
                                        transforms.RandomHorizontalFlip(),
                                        transforms.RandomVerticalFlip(),
                                        transforms.Normalize([0.485, 0.456, 0.406], [0.229, 0.224, 0.225])
                                       ])
#         print(Image.open(self.X[index]))
        image = transform(self.X[index]).float()
        label = self.y[index].astype(float)

        return image, label
    
class MyData_test(Dataset):
    def __init__(self, X, y):
        self.X = X
        self.classes = np.unique(y)
        self.y = np.array(y)
        self.tumorid = y.index

    def __len__(self):
        return len(self.X)

    def __getitem__(self, index):
        transform = transforms.Compose([
                                        transforms.Normalize([0.485, 0.456, 0.406], [0.229, 0.224, 0.225])
                                       ])
        image = transform(torch.tensor(np.moveaxis(np.array(Image.open(self.X[index])), -1, 0).astype(float))).float()
        label = self.y[index].astype(float)

        return image, label
```

In [17]:

```
meta_test = meta_nondup[meta_nondup['Center']=='01']
meta_train = meta_nondup[meta_nondup['Center']!='01']

eeres_threshold = meta_train["HALLMARK_ESTROGEN_RESPONSE_EARLY"].loc[meta_train.index].median()
meta_train['y'] = (meta_train['HALLMARK_ESTROGEN_RESPONSE_EARLY']>eeres_threshold).apply(lambda x: 1 if x else 0)
meta_test['y'] = (meta_test['HALLMARK_ESTROGEN_RESPONSE_EARLY']>eeres_threshold).apply(lambda x: 1 if x else 0)

print("EERES threshold:", eeres_threshold)

X_train, y_train = (prefix + meta_train["File ID"] + '/' + meta_train["File Name"] + "-1.jpg").values, meta_train['y']
X_test, y_test = (prefix + meta_test["File ID"] + '/' + meta_test["File Name"] + "-1.jpg").values, meta_test['y']

print("Total number of data:", len(meta_train)+len(meta_test))

train_dataset = MyData_train(X_train, y_train)
train_dataloader = DataLoader(train_dataset,batch_size=3,shuffle=True)

data = {}
data['train'] = train_dataloader
data['val'] = train_dataloader
dataset_sizes = {}
dataset_sizes[0] = {'train': len(train_dataset), 'val': len(train_dataset)}

# Testing data with X_test/y_test
print(f"Test data: {len(X_test)}")
test_dataset = MyData_test(X_test, y_test)
test_dataloader = DataLoader(test_dataset,batch_size=1,shuffle=False)
```

```
/tmp/ipykernel_54612/2136148646.py:5: SettingWithCopyWarning: 
A value is trying to be set on a copy of a slice from a DataFrame.
Try using .loc[row_indexer,col_indexer] = value instead

See the caveats in the documentation: https://pandas.pydata.org/pandas-docs/stable/user_guide/indexing.html#returning-a-view-versus-a-copy
  meta_train['y'] = (meta_train['HALLMARK_ESTROGEN_RESPONSE_EARLY']>eeres_threshold).apply(lambda x: 1 if x else 0)
/tmp/ipykernel_54612/2136148646.py:6: SettingWithCopyWarning: 
A value is trying to be set on a copy of a slice from a DataFrame.
Try using .loc[row_indexer,col_indexer] = value instead

See the caveats in the documentation: https://pandas.pydata.org/pandas-docs/stable/user_guide/indexing.html#returning-a-view-versus-a-copy
  meta_test['y'] = (meta_test['HALLMARK_ESTROGEN_RESPONSE_EARLY']>eeres_threshold).apply(lambda x: 1 if x else 0)
```

```
EERES threshold: -0.0375978750354623
Total number of data: 1077
Test data: 812
```

## Training¶

In [18]:

```
folder = "Output21"
!mkdir "Output21"
```

```
mkdir: cannot create directory ‘Output21’: File exists
```

In [19]:

```
# Training function

def train_model(model, criterion, optimizer, scheduler, num_epochs=25, folder=None, folds=None):
    device = torch.device("cuda:0" if torch.cuda.is_available() else "cpu")
    since = time.time()
    checkpoint = torch.load(folder+"/model_initial.pt", map_location=torch.device('cpu'))
    
    for fold in folds:
#         last = np.sort(os.listdir(f"{folder}/{str(fold)}"))[-1]
#         print(last)
#         checkpoint = torch.load(folder+f"/{fold}/{last}", map_location=torch.device('cpu'))
        best_auroc = 0.0
        best_average_loss = 100
        model.load_state_dict(checkpoint['model_state_dict'], strict=False)
        model.to(device)
        optimizer.load_state_dict(checkpoint['optimizer_state_dict'])
        folder_fold = folder+"/"+str(fold)
        !mkdir "{folder_fold}"
        for epoch in range(num_epochs):
            print(f'Epoch {epoch}/{num_epochs - 1}')
            print('-' * 10)

            # Each epoch has a training and validation phase
            train_epoch_loss = 100
            val_epoch_loss = 100
            for phase in ['train', 'val']:
                if phase == 'train':
                    model.train()  # Set model to training mode
                else:
                    model.eval()   # Set model to evaluate mode

                running_loss = 0.0
                running_corrects = 0
                running_scores = []
                running_golds = []
                # Iterate over data.
                for inputs, labels in data[phase]:
#                 for inputs, labels in data[phase]:
                    inputs = inputs.to(device)
                    labels = labels.reshape(-1,1).to(device)

                    # zero the parameter gradients
                    optimizer.zero_grad()

                    # forward
                    # track history if only in train
                    with torch.set_grad_enabled(phase == 'train'):
                        outputs = model(inputs)
                        running_scores += torch.flatten(outputs[:,1]).tolist()
                        running_golds += torch.flatten(labels).tolist()
                        _, preds = torch.max(outputs, 1)
                        preds = torch.reshape(preds, (-1,1)).float()
                        gold = torch.tensor([[1,0] if label==0 else [0,1] for label in labels]).to(device).float()
                        loss = criterion(outputs.float(), gold.float())

                        # backward + optimize only if in training phase
                        if phase == 'train':
                            loss.backward()
                            optimizer.step()

                    # statistics
                    running_loss += loss.item() * inputs.size(0)
                    running_corrects += torch.sum(preds == labels.data)
                if phase == 'train':
                    scheduler.step()

                epoch_loss = running_loss / dataset_sizes[fold][phase]
                epoch_auroc = roc_auc_score(running_golds, running_scores)
    
                if phase == 'train':
                    train_epoch_loss = epoch_loss
                elif phase == 'val':
                    val_epoch_loss = epoch_loss
                    average_loss = (train_epoch_loss+val_epoch_loss)/2
                    print(f'{phase} Average Loss: {average_loss}')
                    
                print(f'{phase} Loss: {epoch_loss:.4f} AUROC {epoch_auroc:.5f}')
                
                # Save the model with highest val AUROC
#                 if phase == 'val' and best_auroc < epoch_auroc:
                if phase == 'val':
                    best_average_loss = average_loss
                    best_auroc = epoch_auroc
                    torch.save({
                    'epoch': epoch,
                    'model_state_dict': model.state_dict(),
                    'optimizer_state_dict': optimizer.state_dict(),
                    'loss': loss,
                    }, folder_fold+"/epoch_"+f'{(epoch):03d}'+"_auroc_"+str(epoch_auroc)[:8]+".pt")

            print()

        time_elapsed = time.time() - since
        print(f'Training complete in {time_elapsed // 60:.0f}m {time_elapsed % 60:.0f}s')
        print(f'Best val AUROC: {best_auroc:4f}')
```

In [20]:

```
device = torch.device("cuda" if torch.cuda.is_available() else "cpu")

# Transfer learning with ResNet50 and followed by 3 fc layers

model_ft = models.resnet101(weights='DEFAULT').to(device)
import torch.nn.functional as F
class net(nn.Module):
    def __init__(self):
        super(net, self).__init__()
        self.fc1 = nn.Linear(1000, 128)
        self.fc2 = nn.Linear(128, 32)
        self.fc3 = nn.Linear(32, 2)
        self.m = nn.Dropout(p=0.2)
    
    def forward(self, x):
        x = self.m(x)
        x = F.relu(self.fc1(x))
        x = self.m(x)
        x = F.relu(self.fc2(x))
        x = self.m(x)
        x = F.softmax(self.fc3(x), dim=1)
        return x

net_add = net()

model_ft = nn.Sequential(model_ft, net_add).to(device)

# Observe that all parameters are being optimized
optimizer_ft = optim.SGD(model_ft.parameters(), lr=0.001, momentum=0.9)

# Decay LR by a factor of 0.1 every 7 epochs
exp_lr_scheduler = lr_scheduler.StepLR(optimizer_ft, step_size=7, gamma=0.1)

# Save initial model parameters for each fold of CV
torch.save({
            'model_state_dict': model_ft.state_dict(),
            'optimizer_state_dict': optimizer_ft.state_dict(),
            }, folder+"/model_initial.pt")

criterion = nn.CrossEntropyLoss()
```

In [282]:

```
train_model(model_ft, criterion, optimizer_ft, exp_lr_scheduler,
                       num_epochs=30, folder=folder, folds=[0])
```

```
Epoch 0/29
----------
train Loss: 0.6939 AUROC 0.46896
val Average Loss: 0.6936677733682236
val Loss: 0.6934 AUROC 0.47340

Epoch 1/29
----------
train Loss: 0.6946 AUROC 0.40453
val Average Loss: 0.6939797193374273
val Loss: 0.6933 AUROC 0.49447

Epoch 2/29
----------
train Loss: 0.6937 AUROC 0.48798
val Average Loss: 0.6934905170269732
val Loss: 0.6933 AUROC 0.48832

Epoch 3/29
----------
train Loss: 0.6927 AUROC 0.52763
val Average Loss: 0.6928979081927605
val Loss: 0.6931 AUROC 0.50422

Epoch 4/29
----------
train Loss: 0.6930 AUROC 0.51441
val Average Loss: 0.6930924925039399
val Loss: 0.6932 AUROC 0.50649

Epoch 5/29
----------
train Loss: 0.6931 AUROC 0.49305
val Average Loss: 0.6929808560407387
val Loss: 0.6929 AUROC 0.54870

Epoch 6/29
----------
train Loss: 0.6933 AUROC 0.49459
val Average Loss: 0.6928734558933186
val Loss: 0.6924 AUROC 0.59917

Epoch 7/29
----------
train Loss: 0.6926 AUROC 0.54095
val Average Loss: 0.692794830956549
val Loss: 0.6930 AUROC 0.51515

Epoch 8/29
----------
train Loss: 0.6939 AUROC 0.44230
val Average Loss: 0.6932963407264565
val Loss: 0.6927 AUROC 0.55645

Epoch 9/29
----------
train Loss: 0.6932 AUROC 0.48815
val Average Loss: 0.6930612090623604
val Loss: 0.6929 AUROC 0.52728

Epoch 10/29
----------
train Loss: 0.6936 AUROC 0.48416
val Average Loss: 0.6931527341311833
val Loss: 0.6927 AUROC 0.57314

Epoch 11/29
----------
train Loss: 0.6927 AUROC 0.52421
val Average Loss: 0.6927138821134027
val Loss: 0.6927 AUROC 0.55628

Epoch 12/29
----------
train Loss: 0.6938 AUROC 0.45380
val Average Loss: 0.6934344299559323
val Loss: 0.6931 AUROC 0.51190

Epoch 13/29
----------
train Loss: 0.6936 AUROC 0.49094
val Average Loss: 0.693142728310711
val Loss: 0.6927 AUROC 0.56915

Epoch 14/29
----------
train Loss: 0.6921 AUROC 0.56061
val Average Loss: 0.6922546722978915
val Loss: 0.6925 AUROC 0.58481

Epoch 15/29
----------
train Loss: 0.6935 AUROC 0.48229
val Average Loss: 0.693001997920702
val Loss: 0.6925 AUROC 0.59193

Epoch 16/29
----------
train Loss: 0.6929 AUROC 0.50034
val Average Loss: 0.6928021677260129
val Loss: 0.6927 AUROC 0.57331

Epoch 17/29
----------
train Loss: 0.6924 AUROC 0.54927
val Average Loss: 0.6925922677202045
val Loss: 0.6928 AUROC 0.55594

Epoch 18/29
----------
train Loss: 0.6920 AUROC 0.57445
val Average Loss: 0.6925020385463283
val Loss: 0.6930 AUROC 0.53008

Epoch 19/29
----------
train Loss: 0.6928 AUROC 0.54420
val Average Loss: 0.6925372448732268
val Loss: 0.6923 AUROC 0.60344

Epoch 20/29
----------
train Loss: 0.6928 AUROC 0.52848
val Average Loss: 0.6928396433029536
val Loss: 0.6929 AUROC 0.54238

Epoch 21/29
----------
train Loss: 0.6935 AUROC 0.48992
val Average Loss: 0.6930308402709242
val Loss: 0.6926 AUROC 0.57171

Epoch 22/29
----------
train Loss: 0.6925 AUROC 0.54323
val Average Loss: 0.692774273314566
val Loss: 0.6931 AUROC 0.53606

Epoch 23/29
----------
train Loss: 0.6925 AUROC 0.54773
val Average Loss: 0.6926754349807523
val Loss: 0.6928 AUROC 0.52085

Epoch 24/29
----------
train Loss: 0.6928 AUROC 0.52187
val Average Loss: 0.6927185940292646
val Loss: 0.6927 AUROC 0.56961

Epoch 25/29
----------
train Loss: 0.6937 AUROC 0.47289
val Average Loss: 0.6933237349087338
val Loss: 0.6930 AUROC 0.54113

Epoch 26/29
----------
train Loss: 0.6939 AUROC 0.46229
val Average Loss: 0.6934024318209234
val Loss: 0.6929 AUROC 0.53776

Epoch 27/29
----------
train Loss: 0.6917 AUROC 0.59712
val Average Loss: 0.6920814859417249
val Loss: 0.6925 AUROC 0.58117

Epoch 28/29
----------
train Loss: 0.6925 AUROC 0.54101
val Average Loss: 0.6925850642177294
val Loss: 0.6927 AUROC 0.55035

Epoch 29/29
----------
train Loss: 0.6927 AUROC 0.53156
val Average Loss: 0.6926227074749065
val Loss: 0.6925 AUROC 0.58675

Training complete in 16m 57s
Best val AUROC: 0.586751
```

## Evaluation with unseen testing data¶

In [21]:

```
import torch.nn.functional as F
cv_test_index = y_test.index
cv_predicted_df_dict = {}
folds = [0]
for ep in np.arange(29,30,1):
    print(ep)
    for fold in folds:
        cv_test_predicted_df = pd.DataFrame(np.zeros((len(cv_test_index),2)), index=cv_test_index, columns=['score','gold'])
        file = np.sort(os.listdir(f"{folder}/{str(fold)}"))[ep] # Get the last model file in the folder
        print(f"Fold {fold} model:", file)
        model_path = f"{folder}/{str(fold)}/{file}"
        checkpoint = torch.load(model_path, map_location=torch.device('cpu'))
        model_ft = models.resnet101().to(device)
        model_test = nn.Sequential(model_ft, net_add).to(device)
        model_test.load_state_dict(checkpoint['model_state_dict'])
        model_test.cuda()
        criterion = nn.CrossEntropyLoss()
        epoch_test = checkpoint['epoch']
        loss_test = checkpoint['loss']
        model_test.eval()

        loss_epoch_test=[]
        y_proba = []
        y_gold = []
        y_pred = []
        with torch.no_grad():
            for b, (X, y) in enumerate(test_dataloader):
                outputs = model_test(X.cuda())
                _, preds = torch.max(outputs, 1)
                y_proba += torch.flatten(outputs[:,1]).cpu().tolist()
                y_gold += y.data.cpu().tolist()
                y_pred += preds.cpu().tolist()
    #             loss = criterion(outputs.float(), torch.tensor([[1,0] if label==0 else [0,1] for label in y_test]).to(device).float())
    #             loss_epoch_test.append(loss.item())

            auc=roc_auc_score(y_gold,y_proba)
            print(f"Fold {fold} AUROC: {auc}")
        cv_test_predicted_df.loc[cv_test_index,'score'] = y_proba
        cv_test_predicted_df.loc[cv_test_index,'gold'] = y_gold
        cv_predicted_df_dict[fold] = cv_test_predicted_df

    # Average the scores of the 5 folds
    scores_sum=0
    for f in folds:
        scores_sum += cv_predicted_df_dict[f]["score"]
    cv_test_predicted_df["score"] = scores_sum/len(folds)
    
    # cv_test_predicted_df["score"] = cv_predicted_df_dict[0]["score"]
    cv_test_predicted_df_ihc = cv_test_predicted_df.join(meta_erihc, how='inner')
    cv_test_predicted_df_ihc["Subtype"] = None
    cv_test_predicted_df_ihc["Subtype"][(cv_test_predicted_df_ihc["er_status_by_ihc"]=='Positive') & (cv_test_predicted_df_ihc["her2_status_by_ihc"]=='Negative')] = "ER+/HER2-"
    # cv_predicted_df_ihc["Subtype"][cv_predicted_df_ihc["her2_status_by_ihc"]=='Positive'] = "ERBB2+"
    cv_test_predicted_df_ihc["Subtype"][(cv_test_predicted_df_ihc["er_status_by_ihc"]=='Negative') & (cv_test_predicted_df_ihc["her2_status_by_ihc"]=='Negative') & (cv_test_predicted_df_ihc["pr_status_by_ihc"]=='Negative')] = "TNBC"
    cv_test_predicted_df_ihc["EERES"] = brca_earlyes_es.loc[cv_test_predicted_df_ihc.index]
    cv_test_predicted_df_ihc["ESR1"] = brca_lv3.loc[cv_test_predicted_df_ihc.index]["ESR1"]

    brca_clinical_testing = brca_clinical.join(cv_test_predicted_df_ihc, how='inner')
    brca_clinical_testing_dfs = brca_clinical_testing[["DFS_MONTHS", "DFS_STATUS", "score", "Subtype", "EERES", "ESR1"]].dropna()
    brca_clinical_testing_dss = brca_clinical_testing[["DSS_MONTHS", "DSS_STATUS", "score", "Subtype", "EERES", "ESR1"]].dropna()
    brca_clinical_testing_pfs = brca_clinical_testing[["PFS_MONTHS", "PFS_STATUS", "score", "Subtype", "EERES", "ESR1"]].dropna()
    brca_clinical_testing_os = brca_clinical_testing[["OS_MONTHS", "OS_STATUS", "score", "Subtype", "EERES", "ESR1"]].dropna()

    plt.scatter(cv_test_predicted_df_ihc["EERES"], cv_test_predicted_df_ihc["score"])
    plt.xlabel("EERES")
    plt.ylabel("Score")
    r, p = pearsonr(cv_test_predicted_df_ihc["EERES"], cv_test_predicted_df_ihc["score"])
    plt.title(f"Spearman R: {r}, p-value: {p}")

    auroc = roc_auc_score(cv_test_predicted_df_ihc["gold"], cv_test_predicted_df_ihc["score"])
    RocCurveDisplay.from_predictions(
        cv_test_predicted_df_ihc["gold"],
        cv_test_predicted_df_ihc["score"],
        name=f"EERES>{eeres_threshold:.3f}",
        color="darkorange",
    )
    # plt.plot([0, 1], [0, 1], "k--", label="chance level (AUC = 0.5)")
    plt.axis("square")
    plt.xlabel("False Positive Rate")
    plt.ylabel("True Positive Rate")
    # plt.title("Receiver Operating Characteristic")
    plt.legend()
    plt.show()

    brca_clinical_testing_erposerbb2neg = brca_clinical.join(cv_test_predicted_df_ihc[cv_test_predicted_df_ihc['Subtype']=="ER+/HER2-"], how='inner')
    brca_clinical_testing_erposerbb2neg

    brca_clinical_testing_erposerbb2neg_dfs = brca_clinical_testing_erposerbb2neg[["DFS_MONTHS", "DFS_STATUS", "score", "EERES", "ESR1"]].dropna()
    brca_clinical_testing_erposerbb2neg_dss = brca_clinical_testing_erposerbb2neg[["DSS_MONTHS", "DSS_STATUS", "score", "EERES", "ESR1"]].dropna()
    brca_clinical_testing_erposerbb2neg_pfs = brca_clinical_testing_erposerbb2neg[["PFS_MONTHS", "PFS_STATUS", "score", "EERES", "ESR1"]].dropna()
    brca_clinical_testing_erposerbb2neg_os = brca_clinical_testing_erposerbb2neg[["OS_MONTHS", "OS_STATUS", "score", 'EERES', 'ESR1']].dropna()

    finding_best_threshold_with_FS_SS(brca_clinical_testing_erposerbb2neg_dfs, brca_clinical_testing_erposerbb2neg_dss, ["DFS", "DSS"], 'score', brca_clinical_testing_erposerbb2neg_dss['score'].quantile(0.1), brca_clinical_testing_erposerbb2neg_dss['score'].quantile(0.9), "Predicted Score")
    finding_best_threshold_with_FS_SS(brca_clinical_testing_erposerbb2neg_pfs, brca_clinical_testing_erposerbb2neg_os, ["PFS", "OS"], 'score', brca_clinical_testing_erposerbb2neg_os['score'].quantile(0.1), brca_clinical_testing_erposerbb2neg_os['score'].quantile(0.9), "Predicted Score")
    
    brca_clinical_score = brca_clinical.join(cv_test_predicted_df_ihc, how='inner')[['PFS_MONTHS','PFS_STATUS','ESR1','score','age_at_diagnosis','ajcc_pathologic_tumor_stage']].dropna()
    cph_pfs = CoxPHFitter()
    cph_pfs.fit(brca_clinical_score[['PFS_MONTHS','PFS_STATUS','score']], duration_col='PFS_MONTHS', event_col='PFS_STATUS')

    cph_pfs.print_summary()  # access the individual results using cph.summary
```

```
29
Fold 0 model: epoch_029_auroc_0.586750.pt
Fold 0 AUROC: 0.5348876647970303
```

```
/tmp/ipykernel_54612/1897047101.py:51: SettingWithCopyWarning: 
A value is trying to be set on a copy of a slice from a DataFrame

See the caveats in the documentation: https://pandas.pydata.org/pandas-docs/stable/user_guide/indexing.html#returning-a-view-versus-a-copy
  cv_test_predicted_df_ihc["Subtype"][(cv_test_predicted_df_ihc["er_status_by_ihc"]=='Positive') & (cv_test_predicted_df_ihc["her2_status_by_ihc"]=='Negative')] = "ER+/HER2-"
/tmp/ipykernel_54612/1897047101.py:53: SettingWithCopyWarning: 
A value is trying to be set on a copy of a slice from a DataFrame

See the caveats in the documentation: https://pandas.pydata.org/pandas-docs/stable/user_guide/indexing.html#returning-a-view-versus-a-copy
  cv_test_predicted_df_ihc["Subtype"][(cv_test_predicted_df_ihc["er_status_by_ihc"]=='Negative') & (cv_test_predicted_df_ihc["her2_status_by_ihc"]=='Negative') & (cv_test_predicted_df_ihc["pr_status_by_ihc"]=='Negative')] = "TNBC"
```

```
not significant
not significant
```

```
/home/oscar/.local/lib/python3.10/site-packages/lifelines/utils/__init__.py:1187: UserWarning: Attempting to convert an unexpected datatype 'object' to float. Suggestion: 1) use `lifelines.utils.datetimes_to_durations` to do conversions or 2) manually convert to floats/booleans.
  warnings.warn(warning_text, UserWarning)
/home/oscar/.local/lib/python3.10/site-packages/lifelines/utils/__init__.py:1102: ConvergenceWarning: Column(s) ['score'] have very low variance. This may harm convergence. 1) Are you using formula's? Did you mean to add '-1' to the end. 2) Try dropping this redundant column before fitting if convergence fails.

  warnings.warn(dedent(warning_text), ConvergenceWarning)
```

|  |  |
| --- | --- |
| model | lifelines.CoxPHFitter |
| duration col | 'PFS\_MONTHS' |
| event col | 'PFS\_STATUS' |
| baseline estimation | breslow |
| number of observations | 810 |
| number of events observed | 810 |
| partial log-likelihood | -4614.22 |
| time fit was run | 2023-11-08 17:35:15 UTC |

|  | coef | exp(coef) | se(coef) | coef lower 95% | coef upper 95% | exp(coef) lower 95% | exp(coef) upper 95% | cmp to | z | p | -log2(p) |
| --- | --- | --- | --- | --- | --- | --- | --- | --- | --- | --- | --- |
| score | -23.16 | 0.00 | 7.60 | -38.05 | -8.28 | 0.00 | 0.00 | 0.00 | -3.05 | <0.005 | 8.77 |

  

|  |  |
| --- | --- |
| Concordance | 0.53 |
| Partial AIC | 9230.44 |
| log-likelihood ratio test | 9.29 on 1 df |
| -log2(p) of ll-ratio test | 8.76 |

## Examine the Survival of the testing data (ER+/HER2- vs TNBC)¶

In [22]:

```
cv_test_predicted_df_ihc = cv_test_predicted_df.join(meta_erihc, how='inner')
cv_test_predicted_df_ihc["Subtype"] = None
cv_test_predicted_df_ihc["Subtype"][(cv_test_predicted_df_ihc["er_status_by_ihc"]=='Positive') & (cv_test_predicted_df_ihc["her2_status_by_ihc"]=='Negative')] = "ER+/HER2-"
# cv_predicted_df_ihc["Subtype"][cv_predicted_df_ihc["her2_status_by_ihc"]=='Positive'] = "ERBB2+"
cv_test_predicted_df_ihc["Subtype"][(cv_test_predicted_df_ihc["er_status_by_ihc"]=='Negative') & (cv_test_predicted_df_ihc["her2_status_by_ihc"]=='Negative') & (cv_test_predicted_df_ihc["pr_status_by_ihc"]=='Negative')] = "TNBC"
cv_test_predicted_df_ihc["EERES"] = brca_earlyes_es.loc[cv_test_predicted_df_ihc.index]
cv_test_predicted_df_ihc["ESR1"] = brca_lv3.loc[cv_test_predicted_df_ihc.index]["ESR1"]

brca_clinical_testing = brca_clinical.join(cv_test_predicted_df_ihc, how='inner')
brca_clinical_testing_dfs = brca_clinical_testing[["DFS_MONTHS", "DFS_STATUS", "score", "Subtype", "EERES", "ESR1"]].dropna()
brca_clinical_testing_dss = brca_clinical_testing[["DSS_MONTHS", "DSS_STATUS", "score", "Subtype", "EERES", "ESR1"]].dropna()
brca_clinical_testing_pfs = brca_clinical_testing[["PFS_MONTHS", "PFS_STATUS", "score", "Subtype", "EERES", "ESR1"]].dropna()
brca_clinical_testing_os = brca_clinical_testing[["OS_MONTHS", "OS_STATUS", "score", "Subtype", "EERES", "ESR1"]].dropna()

result = km.fit(brca_clinical_testing_pfs['PFS_MONTHS'], brca_clinical_testing_pfs['PFS_STATUS'], brca_clinical_testing_pfs["Subtype"])
km.plot(result, title=f"PFS of ER+/HER2- vs TNBC, Logrank p-value={result['logrank_P']:.3e}", full_ylim=True, y_percentage=True)
plt.show()
result = km.fit(brca_clinical_testing_os['OS_MONTHS'], brca_clinical_testing_os['OS_STATUS'], brca_clinical_testing_os["Subtype"])
km.plot(result, title=f"OS of ER+/HER2- vs TNBC, Logrank p-value={result['logrank_P']:.3e}", full_ylim=True, y_percentage=True)
plt.show()
result = km.fit(brca_clinical_testing_dfs['DFS_MONTHS'], brca_clinical_testing_dfs['DFS_STATUS'], brca_clinical_testing_dfs["Subtype"])
km.plot(result, title=f"DFS of ER+/HER2- vs TNBC, Logrank p-value={result['logrank_P']:.3e}", full_ylim=True, y_percentage=True)
plt.show()
result = km.fit(brca_clinical_testing_dss['DSS_MONTHS'], brca_clinical_testing_dss['DSS_STATUS'], brca_clinical_testing_dss["Subtype"])
km.plot(result, title=f"DSS of ER+/HER2- vs TNBC, Logrank p-value={result['logrank_P']:.3e}", full_ylim=True, y_percentage=True)
plt.show()


plt.scatter(cv_test_predicted_df_ihc[cv_test_predicted_df_ihc["Subtype"]=="ER+/HER2-"]["ESR1"].apply(math.log), cv_test_predicted_df_ihc[cv_test_predicted_df_ihc["Subtype"]=="ER+/HER2-"]["EERES"])
# plt.scatter(cv_predicted_df_ihc[cv_predicted_df_ihc["Subtype"]=="ERBB2+"]["esr1"].apply(math.log), cv_predicted_df_ihc[cv_predicted_df_ihc["Subtype"]=="ERBB2+"]["eeres"])
plt.scatter(cv_test_predicted_df_ihc[cv_test_predicted_df_ihc["Subtype"]=="TNBC"]["ESR1"].apply(math.log), cv_test_predicted_df_ihc[cv_test_predicted_df_ihc["Subtype"]=="TNBC"]["EERES"])
plt.legend(["ER+/HER2-", "TNBC"])
plt.xlabel("ESR1")
plt.ylabel("EERES")
plt.show()
```

```
/tmp/ipykernel_54612/4221478194.py:3: SettingWithCopyWarning: 
A value is trying to be set on a copy of a slice from a DataFrame

See the caveats in the documentation: https://pandas.pydata.org/pandas-docs/stable/user_guide/indexing.html#returning-a-view-versus-a-copy
  cv_test_predicted_df_ihc["Subtype"][(cv_test_predicted_df_ihc["er_status_by_ihc"]=='Positive') & (cv_test_predicted_df_ihc["her2_status_by_ihc"]=='Negative')] = "ER+/HER2-"
/tmp/ipykernel_54612/4221478194.py:5: SettingWithCopyWarning: 
A value is trying to be set on a copy of a slice from a DataFrame

See the caveats in the documentation: https://pandas.pydata.org/pandas-docs/stable/user_guide/indexing.html#returning-a-view-versus-a-copy
  cv_test_predicted_df_ihc["Subtype"][(cv_test_predicted_df_ihc["er_status_by_ihc"]=='Negative') & (cv_test_predicted_df_ihc["her2_status_by_ihc"]=='Negative') & (cv_test_predicted_df_ihc["pr_status_by_ihc"]=='Negative')] = "TNBC"
```

In [23]:

```
print('Whole samples')
plt.scatter(cv_test_predicted_df_ihc["EERES"], cv_test_predicted_df_ihc["score"])
plt.xlabel("EERES")
plt.ylabel("Score")
r, p = pearsonr(cv_test_predicted_df_ihc["EERES"], cv_test_predicted_df_ihc["score"])
plt.title(f"Pearson R: {r}, p-value: {p}")
plt.show()


print('ER+/HER2- samples')
plt.scatter(cv_test_predicted_df_ihc[cv_test_predicted_df_ihc['Subtype']=="ER+/HER2-"]["EERES"], cv_test_predicted_df_ihc[cv_test_predicted_df_ihc['Subtype']=="ER+/HER2-"]["score"])
plt.xlabel("EERES")
plt.ylabel("Score")
r, p = pearsonr(cv_test_predicted_df_ihc[cv_test_predicted_df_ihc['Subtype']=="ER+/HER2-"]["EERES"], cv_test_predicted_df_ihc[cv_test_predicted_df_ihc['Subtype']=="ER+/HER2-"]["score"])
plt.title(f"Pearson R: {r}, p-value: {p}")
plt.show() # Figure 3a

print('TNBC samples')
plt.scatter(cv_test_predicted_df_ihc[cv_test_predicted_df_ihc['Subtype']=="TNBC"]["EERES"], cv_test_predicted_df_ihc[cv_test_predicted_df_ihc['Subtype']=="TNBC"]["score"])
plt.xlabel("EERES")
plt.ylabel("Score")
r, p = pearsonr(cv_test_predicted_df_ihc[cv_test_predicted_df_ihc['Subtype']=="TNBC"]["EERES"], cv_test_predicted_df_ihc[cv_test_predicted_df_ihc['Subtype']=="TNBC"]["score"])
plt.title(f"Pearson R: {r}, p-value: {p}")
plt.show()

print('Others')
q = (cv_test_predicted_df_ihc['Subtype']=="ER+/HER2-") | (cv_test_predicted_df_ihc['Subtype']=="TNBC")

plt.scatter(cv_test_predicted_df_ihc[~q]["EERES"], cv_test_predicted_df_ihc[~q]["score"])
plt.xlabel("EERES")
plt.ylabel("Score")
r, p = pearsonr(cv_test_predicted_df_ihc[q]["EERES"], cv_test_predicted_df_ihc[q]["score"])
plt.title(f"Pearson R: {r}, p-value: {p}")
plt.show()
```

```
Whole samples
```

```
ER+/HER2- samples
```

```
TNBC samples
```

```
Others
```

In [24]:

```
brca_clinical_testing_erposerbb2neg = brca_clinical.join(cv_test_predicted_df_ihc[cv_test_predicted_df_ihc['Subtype']=="ER+/HER2-"], how='inner')
brca_clinical_testing_erposerbb2neg

brca_clinical_testing_erposerbb2neg_dfs = brca_clinical_testing_erposerbb2neg[["DFS_MONTHS", "DFS_STATUS", "score", "EERES", "ESR1"]].dropna()
brca_clinical_testing_erposerbb2neg_dss = brca_clinical_testing_erposerbb2neg[["DSS_MONTHS", "DSS_STATUS", "score", "EERES", "ESR1"]].dropna()
brca_clinical_testing_erposerbb2neg_pfs = brca_clinical_testing_erposerbb2neg[["PFS_MONTHS", "PFS_STATUS", "score", "EERES", "ESR1"]].dropna()
brca_clinical_testing_erposerbb2neg_os = brca_clinical_testing_erposerbb2neg[["OS_MONTHS", "OS_STATUS", "score", 'EERES', 'ESR1']].dropna()
# brca_clinical_testing_erposerbb2neg_dfs = brca_clinical_testing_erposerbb2neg_dfs[brca_clinical_testing_erposerbb2neg_dfs['DFS_STATUS']=='1']
# brca_clinical_testing_erposerbb2neg_dss = brca_clinical_testing_erposerbb2neg_dss[brca_clinical_testing_erposerbb2neg_dss['DSS_STATUS']=='1']
# brca_clinical_testing_erposerbb2neg_pfs = brca_clinical_testing_erposerbb2neg_pfs[brca_clinical_testing_erposerbb2neg_pfs['PFS_STATUS']=='1']
# brca_clinical_testing_erposerbb2neg_os = brca_clinical_testing_erposerbb2neg_os[brca_clinical_testing_erposerbb2neg_os['OS_STATUS']=='1']
```

In [40]:

```
sss
```

Out[40]:

```
TCGA-3C-AAAU    0.495800
TCGA-5L-AAT0    0.501751
TCGA-A1-A0SB    0.497689
TCGA-A1-A0SD    0.493312
TCGA-A1-A0SE    0.488574
                  ...   
TCGA-PE-A5DE    0.496955
TCGA-S3-AA12    0.490890
TCGA-WT-AB44    0.496316
TCGA-XX-A89A    0.489651
TCGA-Z7-A8R6    0.496626
Name: score, Length: 325, dtype: float64
```

## Examine the Survival of the ER+/HER2- testing data (Higher/Lower Predicted Score)¶

In [47]:

```
sss = brca_clinical.join(cv_test_predicted_df_ihc[cv_test_predicted_df_ihc['Subtype']=='ER+/HER2-'],how='inner')['score']

quantile_tested = []
scores_tested = []
p_values_tested = []

for q in np.arange(0.1,0.9,0.01):
    score = sss.quantile(q)
    pred = pd.DataFrame(index=sss.index,columns=['Prediction'])
    pred.loc[sss>=score,'Prediction'] = 'Higher predicted score'
    pred.loc[sss<score,'Prediction'] = 'Lower predicted score'
    result = km.fit(brca_clinical.join(cv_test_predicted_df_ihc[cv_test_predicted_df_ihc['Subtype']=='ER+/HER2-'], how='inner')[f'PFS_MONTHS'], brca_clinical.join(cv_test_predicted_df_ihc[cv_test_predicted_df_ihc['Subtype']=='ER+/HER2-'], how='inner')[f'PFS_STATUS'], pred['Prediction'])
    quantile_tested.append(q)
    scores_tested.append(score)
    p_values_tested.append(result['logrank_P'])
    
# Table S4    
pd.DataFrame({'score':scores_tested,'logrank_p_value':p_values_tested},index=quantile_tested).to_csv('Table S4.csv')

q=0.2
score = sss.quantile(q)
print(q, score)
pred = pd.DataFrame(index=sss.index,columns=['Prediction'])
pred.loc[sss>=score,'Prediction'] = 'Higher predicted score'
pred.loc[sss<score,'Prediction'] = 'Lower predicted score'
result = km.fit(brca_clinical.join(cv_test_predicted_df_ihc[cv_test_predicted_df_ihc['Subtype']=='ER+/HER2-'], how='inner')[f'PFS_MONTHS'], brca_clinical.join(cv_test_predicted_df_ihc[cv_test_predicted_df_ihc['Subtype']=='ER+/HER2-'], how='inner')[f'PFS_STATUS'], pred['Prediction'])
#     if result['logrank_P']<0.05:
km.plot(result, title=f"PFS of ER+/HER2- with higher predicted score VS TNBC, Logrank p-value={result['logrank_P']:.3e}", full_ylim=True, y_percentage=True)
# Figure 3b
plt.show()
aaa = brca_clinical.join(cv_test_predicted_df_ihc[cv_test_predicted_df_ihc['Subtype']=='ER+/HER2-'], how='inner')[sss<s]
aaa['Subtype'] = 'Higher predicted score'
ttt = brca_clinical.join(cv_test_predicted_df_ihc[cv_test_predicted_df_ihc['Subtype']=='TNBC'], how='inner')
at = pd.concat([aaa,ttt])
result = km.fit(at[f'PFS_MONTHS'], at[f'PFS_STATUS'], at['Subtype'])

km.plot(result, title=f"PFS of ER+/HER2- with higher predicted score VS TNBC, Logrank p-value={result['logrank_P']:.3e}", full_ylim=True, y_percentage=True)
plt.show()

aaa = brca_clinical.join(cv_test_predicted_df_ihc[cv_test_predicted_df_ihc['Subtype']=='ER+/HER2-'], how='inner')[sss>=s]
aaa['Subtype'] = 'Lower predicted score'
ttt = brca_clinical.join(cv_test_predicted_df_ihc[cv_test_predicted_df_ihc['Subtype']=='TNBC'], how='inner')
at = pd.concat([aaa,ttt])
pred.loc[sss>=s,'Prediction'] = 'Higher predicted Score'
pred.loc[sss<s,'Prediction'] = 'Lower predicted score'
result = km.fit(at[f'PFS_MONTHS'], at[f'PFS_STATUS'], at['Subtype'])

km.plot(result, title=f"PFS of ER+/HER2- with higher predicted score VS TNBC, Logrank p-value={result['logrank_P']:.3e}", full_ylim=True, y_percentage=True)
plt.show()
```

```
0.2 0.4896386623382568
```

In [26]:

```
# prepare the predicted scores of the training samples

train_dataset = MyData_test(X_train, y_train)
train_dataloader = DataLoader(train_dataset,batch_size=1,shuffle=False)
cv_train_index = y_train.index
cv_train_predicted_df_dict = {}
import torch.nn.functional as F
for fold in [0]:
    cv_train_predicted_df = pd.DataFrame(np.zeros((len(cv_train_index),2)), index=cv_train_index, columns=['score','gold'])
    file = np.sort(os.listdir(f"{folder}/{str(fold)}"))[14] # Get the last model file in the folder
    print(f"Fold {fold} model:", file)
    model_path = f"{folder}/{str(fold)}/{file}"
    checkpoint = torch.load(model_path, map_location=torch.device('cpu'))
    model_ft = models.resnet101().to(device)
    model_test = nn.Sequential(model_ft, net_add).to(device)
    model_test.load_state_dict(checkpoint['model_state_dict'])
    model_test.cuda()
    criterion = nn.CrossEntropyLoss()
    epoch_test = checkpoint['epoch']
    loss_test = checkpoint['loss']
    model_test.eval()

    loss_epoch_test=[]
    y_proba = []
    y_gold = []
    y_pred = []
    with torch.no_grad():
        for b, (X, y) in enumerate(train_dataloader):
            outputs = model_test(X.cuda())
            _, preds = torch.max(outputs, 1)
            y_proba += torch.flatten(outputs[:,1]).cpu().tolist()
            y_gold += y.data.cpu().tolist()
            y_pred += preds.cpu().tolist()
#             loss = criterion(outputs.float(), torch.tensor([[1,0] if label==0 else [0,1] for label in y_test]).to(device).float())
#             loss_epoch_test.append(loss.item())

        auc=roc_auc_score(y_gold,y_proba)
        print(f"Fold {fold} AUROC: {auc}")
    cv_train_predicted_df.loc[cv_train_index,'score'] = y_proba
    cv_train_predicted_df.loc[cv_train_index,'gold'] = y_gold
    cv_train_predicted_df_dict[fold] = cv_train_predicted_df

# Average the scores of the 5 folds
cv_train_predicted_df["score"] = cv_train_predicted_df_dict[0]["score"]
cv_train_predicted_df_ihc = cv_train_predicted_df.join(meta_erihc, how='inner')
cv_train_predicted_df_ihc["Subtype"] = None
cv_train_predicted_df_ihc["Subtype"][(cv_train_predicted_df_ihc["er_status_by_ihc"]=='Positive') & (cv_train_predicted_df_ihc["her2_status_by_ihc"]=='Negative')] = "ER+/HER2-"
# cv_predicted_df_ihc["Subtype"][cv_predicted_df_ihc["her2_status_by_ihc"]=='Positive'] = "ERBB2+"
cv_train_predicted_df_ihc["Subtype"][(cv_train_predicted_df_ihc["er_status_by_ihc"]=='Negative') & (cv_train_predicted_df_ihc["her2_status_by_ihc"]=='Negative') & (cv_test_predicted_df_ihc["pr_status_by_ihc"]=='Negative')] = "TNBC"
cv_train_predicted_df_ihc["EERES"] = brca_earlyes_es.loc[cv_train_predicted_df_ihc.index]
cv_train_predicted_df_ihc["ESR1"] = brca_lv3.loc[cv_train_predicted_df_ihc.index]["ESR1"]
cv_train_predicted_df_ihc
```

```
Fold 0 model: epoch_014_auroc_0.584814.pt
Fold 0 AUROC: 0.5447140578719526
```

```
/tmp/ipykernel_54612/1315662996.py:47: SettingWithCopyWarning: 
A value is trying to be set on a copy of a slice from a DataFrame

See the caveats in the documentation: https://pandas.pydata.org/pandas-docs/stable/user_guide/indexing.html#returning-a-view-versus-a-copy
  cv_train_predicted_df_ihc["Subtype"][(cv_train_predicted_df_ihc["er_status_by_ihc"]=='Positive') & (cv_train_predicted_df_ihc["her2_status_by_ihc"]=='Negative')] = "ER+/HER2-"
/tmp/ipykernel_54612/1315662996.py:49: SettingWithCopyWarning: 
A value is trying to be set on a copy of a slice from a DataFrame

See the caveats in the documentation: https://pandas.pydata.org/pandas-docs/stable/user_guide/indexing.html#returning-a-view-versus-a-copy
  cv_train_predicted_df_ihc["Subtype"][(cv_train_predicted_df_ihc["er_status_by_ihc"]=='Negative') & (cv_train_predicted_df_ihc["her2_status_by_ihc"]=='Negative') & (cv_test_predicted_df_ihc["pr_status_by_ihc"]=='Negative')] = "TNBC"
```

Out[26]:

|  | score | gold | bcr\_patient\_uuid | form\_completion\_date | prospective\_collection | retrospective\_collection | birth\_days\_to | gender | menopause\_status | race | ... | metastatic\_tumor\_indicator | patient\_id | project\_code | site\_of\_primary\_tumor\_other | stage\_other | tissue\_source\_site | tumor\_tissue\_site | Subtype | EERES | ESR1 |
| --- | --- | --- | --- | --- | --- | --- | --- | --- | --- | --- | --- | --- | --- | --- | --- | --- | --- | --- | --- | --- | --- |
| TCGA-BH-A18N | 0.498579 | 1.0 | 665dd3d3-779c-4abd-b5d7-13342340451d | 2011-6-15 | NO | YES | -32404 | FEMALE | [Not Available] | WHITE | ... | NO | A18N | [Not Available] | [Not Applicable] | [Not Available] | BH | Breast | ER+/HER2- | 0.289838 | 33831.2000 |
| TCGA-A2-A0CQ | 0.497316 | 1.0 | ab34a9a2-d72d-4106-94fb-118844b1b60b | 2010-8-10 | NO | YES | -22810 | FEMALE | Post (prior bilateral ovariectomy OR >12 mo si... | BLACK OR AFRICAN AMERICAN | ... | [Not Available] | A0CQ | [Not Available] | [Not Applicable] | [Not Available] | A2 | Breast | None | 0.139648 | 15568.7000 |
| TCGA-A7-A26E | 0.489885 | 1.0 | 011b9b2d-ebe5-42bf-9662-d922faccc7a1 | 2011-7-28 | YES | NO | -26274 | FEMALE | Post (prior bilateral ovariectomy OR >12 mo si... | WHITE | ... | NO | A26E | TCGA | [Not Applicable] | [Not Available] | A7 | Breast | ER+/HER2- | 0.059451 | 24318.8000 |
| TCGA-BH-A18V | 0.501294 | 0.0 | 6b960b58-28e1-41c6-bd6e-7e669c6aa4ef | 2011-7-2 | NO | YES | -17682 | FEMALE | [Not Available] | WHITE | ... | YES | A18V | [Not Available] | [Not Applicable] | [Not Available] | BH | Breast | None | -0.291810 | 154.1140 |
| TCGA-LL-A7T0 | 0.491760 | 0.0 | D8F8064F-02EF-4FED-942B-714CBE5E8455 | 2014-1-3 | YES | NO | -25867 | FEMALE | Post (prior bilateral ovariectomy OR >12 mo si... | BLACK OR AFRICAN AMERICAN | ... | [Not Available] | A7T0 | [Not Available] | [Not Applicable] | [Not Available] | LL | Breast | None | -0.053663 | 10559.3000 |
| ... | ... | ... | ... | ... | ... | ... | ... | ... | ... | ... | ... | ... | ... | ... | ... | ... | ... | ... | ... | ... | ... |
| TCGA-EW-A1OW | 0.488479 | 0.0 | f55dd73d-8c36-440b-84e5-9aae53107775 | 2011-5-18 | NO | YES | -21465 | FEMALE | Post (prior bilateral ovariectomy OR >12 mo si... | BLACK OR AFRICAN AMERICAN | ... | NO | A1OW | [Not Available] | [Not Applicable] | [Not Available] | EW | Breast | None | -0.376722 | 16.7966 |
| TCGA-S3-AA10 | 0.502417 | 0.0 | 23C31C2E-336C-4878-A476-CF8D811B4875 | 2014-4-18 | YES | NO | -24075 | FEMALE | Post (prior bilateral ovariectomy OR >12 mo si... | BLACK OR AFRICAN AMERICAN | ... | [Not Available] | AA10 | [Not Available] | [Not Applicable] | [Not Available] | S3 | Breast | None | -0.306525 | 67.2054 |
| TCGA-AQ-A1H3 | 0.492295 | 1.0 | 82ec33dd-e783-4c74-9a87-797a699e11df | 2011-4-20 | YES | NO | -18177 | FEMALE | Pre (<6 months since LMP AND no prior bilatera... | WHITE | ... | NO | A1H3 | [Not Available] | [Not Applicable] | [Not Available] | AQ | Breast | ER+/HER2- | 0.214703 | 14058.3000 |
| TCGA-A1-A0SO | 0.499182 | 0.0 | 6644fd4e-d2fe-4785-a73c-0f36fcc740e2 | 2010-12-6 | NO | YES | -24826 | FEMALE | Post (prior bilateral ovariectomy OR >12 mo si... | WHITE | ... | [Not Available] | A0SO | [Not Available] | [Not Applicable] | [Not Available] | A1 | Breast | None | -0.427218 | 14.5991 |
| TCGA-E9-A3X8 | 0.496471 | 1.0 | 95873E61-AFDB-496C-9F77-3F9BEB008CDA | 2012-8-24 | YES | NO | -17588 | FEMALE | Post (prior bilateral ovariectomy OR >12 mo si... | WHITE | ... | NO | A3X8 | [Not Available] | [Not Applicable] | [Not Available] | E9 | Breast | None | 0.117333 | 3467.8000 |

265 rows × 116 columns

In [50]:

```
# Figure 4a
from lifelines import CoxPHFitter
brca_clinical_score = brca_clinical.join(cv_test_predicted_df_ihc, how='inner')[['PFS_MONTHS','PFS_STATUS','ESR1','score','age_at_diagnosis','ajcc_pathologic_tumor_stage']].dropna()

brca_clinical_score = brca_clinical_score[['PFS_MONTHS','PFS_STATUS','score','age_at_diagnosis','ajcc_pathologic_tumor_stage']]
cph_pfs_test = CoxPHFitter()
cph_pfs_test.fit(brca_clinical_score, duration_col='PFS_MONTHS', event_col='PFS_STATUS')

cph_pfs_test.summary
```

```
/home/oscar/.local/lib/python3.10/site-packages/lifelines/utils/__init__.py:1187: UserWarning: Attempting to convert an unexpected datatype 'object' to float. Suggestion: 1) use `lifelines.utils.datetimes_to_durations` to do conversions or 2) manually convert to floats/booleans.
  warnings.warn(warning_text, UserWarning)
/home/oscar/.local/lib/python3.10/site-packages/lifelines/utils/__init__.py:1102: ConvergenceWarning: Column(s) ['score'] have very low variance. This may harm convergence. 1) Are you using formula's? Did you mean to add '-1' to the end. 2) Try dropping this redundant column before fitting if convergence fails.

  warnings.warn(dedent(warning_text), ConvergenceWarning)
```

Out[50]:

|  | coef | exp(coef) | se(coef) | coef lower 95% | coef upper 95% | exp(coef) lower 95% | exp(coef) upper 95% | cmp to | z | p | -log2(p) |
| --- | --- | --- | --- | --- | --- | --- | --- | --- | --- | --- | --- |
| covariate |  |  |  |  |  |  |  |  |  |  |  |
| score | -24.137515 | 3.290109e-11 | 7.620636 | -39.073688 | -9.201343 | 1.072786e-17 | 0.000101 | 0.0 | -3.167389 | 0.001538 | 9.344591 |
| age\_at\_diagnosis | 0.012543 | 1.012622e+00 | 0.002651 | 0.007347 | 0.017740 | 1.007374e+00 | 1.017898 | 0.0 | 4.730782 | 0.000002 | 18.770285 |
| ajcc\_pathologic\_tumor\_stage | 0.181208 | 1.198665e+00 | 0.047054 | 0.088984 | 0.273432 | 1.093063e+00 | 1.314468 | 0.0 | 3.851073 | 0.000118 | 13.053809 |

In [51]:

```
from lifelines import CoxPHFitter
brca_clinical_score = brca_clinical.join(cv_train_predicted_df_ihc, how='inner')[['PFS_MONTHS','PFS_STATUS','ESR1','EERES','score','age_at_diagnosis','ajcc_pathologic_tumor_stage']].dropna()
cph_pfs_train = CoxPHFitter()
cph_pfs_train.fit(brca_clinical_score[['PFS_MONTHS','PFS_STATUS','score','age_at_diagnosis','ajcc_pathologic_tumor_stage']], duration_col='PFS_MONTHS', event_col='PFS_STATUS')

cph_pfs_train.summary
```

```
/home/oscar/.local/lib/python3.10/site-packages/lifelines/utils/__init__.py:1187: UserWarning: Attempting to convert an unexpected datatype 'object' to float. Suggestion: 1) use `lifelines.utils.datetimes_to_durations` to do conversions or 2) manually convert to floats/booleans.
  warnings.warn(warning_text, UserWarning)
/home/oscar/.local/lib/python3.10/site-packages/lifelines/utils/__init__.py:1102: ConvergenceWarning: Column(s) ['score'] have very low variance. This may harm convergence. 1) Are you using formula's? Did you mean to add '-1' to the end. 2) Try dropping this redundant column before fitting if convergence fails.

  warnings.warn(dedent(warning_text), ConvergenceWarning)
```

Out[51]:

|  | coef | exp(coef) | se(coef) | coef lower 95% | coef upper 95% | exp(coef) lower 95% | exp(coef) upper 95% | cmp to | z | p | -log2(p) |
| --- | --- | --- | --- | --- | --- | --- | --- | --- | --- | --- | --- |
| covariate |  |  |  |  |  |  |  |  |  |  |  |
| score | -29.744736 | 1.207884e-13 | 11.656509 | -52.591073 | -6.898399 | 1.445396e-23 | 0.001009 | 0.0 | -2.551771 | 0.010718 | 6.543860 |
| age\_at\_diagnosis | 0.006309 | 1.006329e+00 | 0.005019 | -0.003528 | 0.016145 | 9.964785e-01 | 1.016276 | 0.0 | 1.257039 | 0.208739 | 2.260225 |
| ajcc\_pathologic\_tumor\_stage | 0.268421 | 1.307898e+00 | 0.088414 | 0.095132 | 0.441710 | 1.099804e+00 | 1.555365 | 0.0 | 3.035944 | 0.002398 | 8.704048 |

In [66]:

```
aaa = brca_clinical.join(cv_test_predicted_df_ihc[cv_test_predicted_df_ihc['Subtype']=='ER+/HER2-'], how='inner')[['OS_MONTHS','OS_STATUS','PFS_MONTHS','PFS_STATUS','score','age_at_diagnosis','ajcc_pathologic_tumor_stage']].dropna()
sss = cph_pfs_train.predict_partial_hazard(aaa[['PFS_MONTHS','PFS_STATUS','score','age_at_diagnosis','ajcc_pathologic_tumor_stage']].dropna())
quantile_tested = []
scores_tested = []
p_values_tested = []

for q in np.arange(0.1,0.91,0.01):
    score = sss.quantile(q)
    quantile_tested.append(q)
    scores_tested.append(score)
    
    score = sss.quantile(q)
#     print(q, score)
    sss_ = sss.apply(lambda s: 'Higher predicted risk' if s>=score else 'Lower predicted risk')
    
    result = km.fit(aaa[f'PFS_MONTHS'], aaa[f'PFS_STATUS'], sss_)
    p_values_tested.append(result['logrank_P'])
#     if result['logrank_P']<0.05:
#         km.plot(result, title=f"PFS of ER+/HER2- with higher predicted risk VS TNBC, Logrank p-value={result['logrank_P']:.3e}", full_ylim=True, y_percentage=True)
#         plt.show()

pd.DataFrame({'predicted_risk':scores_tested,'logrank_pvalue':p_values_tested},index=quantile_tested).to_csv('Table S5.csv')

q=0.89
score = sss.quantile(q)
print(q, score)
sss_ = sss.apply(lambda s: 'Higher predicted risk' if s>=score else 'Lower predicted risk')

result = km.fit(aaa[f'PFS_MONTHS'], aaa[f'PFS_STATUS'], sss_)
if result['logrank_P']<0.05:
    km.plot(result, title=f"PFS of ER+/HER2- with higher vs lower predicted risk, Logrank p-value={result['logrank_P']:.3e}", full_ylim=True, y_percentage=True)
    plt.show() # Figure 4b
```

```
0.89 1.4964616837445803
```

In [67]:

```
aaa = brca_clinical.join(cv_test_predicted_df_ihc[cv_test_predicted_df_ihc['Subtype']=='ER+/HER2-'], how='inner')[['OS_MONTHS','OS_STATUS','PFS_MONTHS','PFS_STATUS','score','age_at_diagnosis','ajcc_pathologic_tumor_stage']].dropna()
sss = cph_pfs_train.predict_partial_hazard(aaa[['OS_MONTHS','OS_STATUS','age_at_diagnosis','ajcc_pathologic_tumor_stage','score']].dropna())
sss = sss.apply(lambda s: 'Higher predicted risk' if s>=1.49646168374458 else 'Lower predicted risk')
result = km.fit(aaa[f'OS_MONTHS'], aaa[f'OS_STATUS'], sss)
if result['logrank_P']<0.05:
    km.plot(result, title=f"OS of ER+/HER2- with higher vs lower predicted risk, Logrank p-value={result['logrank_P']:.3e}", full_ylim=True, y_percentage=True)
    plt.show()
```

In [71]:

```
sss = cph_pfs_train.predict_partial_hazard(brca_clinical.join(cv_test_predicted_df_ihc[cv_test_predicted_df_ihc['Subtype']=='ER+/HER2-'], how='inner')[['PFS_MONTHS','PFS_STATUS','score','age_at_diagnosis','ajcc_pathologic_tumor_stage']].dropna())
s=1.49646168374458
pred = pd.DataFrame(index=sss.index,columns=['Prediction'])
pred.loc[sss>=s,'Prediction'] = 'Higher predicted risk'
pred.loc[sss<s,'Prediction'] = 'Lower predicted risk'
result = km.fit(brca_clinical.join(cv_test_predicted_df_ihc[cv_test_predicted_df_ihc['Subtype']=='ER+/HER2-'], how='inner')[f'PFS_MONTHS'], brca_clinical.join(cv_test_predicted_df_ihc[cv_test_predicted_df_ihc['Subtype']=='ER+/HER2-'], how='inner')[f'PFS_STATUS'], pred['Prediction'])
#     if result['logrank_P']<0.05:
km.plot(result, title=f"PFS of ER+/HER2- with higher vs lower predicted risk, Logrank p-value={result['logrank_P']:.3e}", full_ylim=True, y_percentage=True)
plt.show() # Figure 4b

aaa = brca_clinical.join(cv_test_predicted_df_ihc[cv_test_predicted_df_ihc['Subtype']=='ER+/HER2-'], how='inner')[sss<s]
aaa['Subtype'] = 'Lower predicted risk'
ttt = brca_clinical.join(cv_test_predicted_df_ihc[cv_test_predicted_df_ihc['Subtype']=='TNBC'], how='inner')
at = pd.concat([aaa,ttt])
result = km.fit(at[f'PFS_MONTHS'], at[f'PFS_STATUS'], at['Subtype'])
#     if result['logrank_P']<0.05:
km.plot(result, title=f"PFS of ER+/HER2- with lower predicted risk VS TNBC, Logrank p-value={result['logrank_P']:.3e}", full_ylim=True, y_percentage=True)
plt.show() # Figure 4d

aaa = brca_clinical.join(cv_test_predicted_df_ihc[cv_test_predicted_df_ihc['Subtype']=='ER+/HER2-'], how='inner')[sss>=s]
aaa['Subtype'] = 'Higher predicted risk'
ttt = brca_clinical.join(cv_test_predicted_df_ihc[cv_test_predicted_df_ihc['Subtype']=='TNBC'], how='inner')
at = pd.concat([aaa,ttt])
result = km.fit(at[f'PFS_MONTHS'], at[f'PFS_STATUS'], at['Subtype'])
#     if result['logrank_P']<0.05:
km.plot(result, title=f"PFS of ER+/HER2- with higher predicted risk VS TNBC, Logrank p-value={result['logrank_P']:.3e}", full_ylim=True, y_percentage=True)
plt.show() # Figure 4f
```

In [72]:

```
sss = cph_pfs_train.predict_partial_hazard(brca_clinical.join(cv_test_predicted_df_ihc[cv_test_predicted_df_ihc['Subtype']=='ER+/HER2-'], how='inner')[['PFS_MONTHS','PFS_STATUS','score','age_at_diagnosis','ajcc_pathologic_tumor_stage']].dropna())
s=1.49646168374458
pred = pd.DataFrame(index=sss.index,columns=['Prediction'])
pred.loc[sss>=s,'Prediction'] = 'Higher predicted risk'
pred.loc[sss<s,'Prediction'] = 'Lower predicted risk'
result = km.fit(brca_clinical.join(cv_test_predicted_df_ihc[cv_test_predicted_df_ihc['Subtype']=='ER+/HER2-'], how='inner')[f'OS_MONTHS'], brca_clinical.join(cv_test_predicted_df_ihc[cv_test_predicted_df_ihc['Subtype']=='ER+/HER2-'], how='inner')[f'OS_STATUS'], pred['Prediction'])
#     if result['logrank_P']<0.05:
km.plot(result, title=f"OS of ER+/HER2- with higher vs lower predicted risk, Logrank p-value={result['logrank_P']:.3e}", full_ylim=True, y_percentage=True)
plt.show() # Figure 4c

aaa = brca_clinical.join(cv_test_predicted_df_ihc[cv_test_predicted_df_ihc['Subtype']=='ER+/HER2-'], how='inner')[sss<s]
aaa['Subtype'] = 'Lower predicted risk'
ttt = brca_clinical.join(cv_test_predicted_df_ihc[cv_test_predicted_df_ihc['Subtype']=='TNBC'], how='inner')
at = pd.concat([aaa,ttt])
result = km.fit(at[f'OS_MONTHS'], at[f'OS_STATUS'], at['Subtype'])
#     if result['logrank_P']<0.05:
km.plot(result, title=f"OS of ER+/HER2- with lower predicted risk VS TNBC, Logrank p-value={result['logrank_P']:.3e}", full_ylim=True, y_percentage=True)
plt.show() # Figure 4e

aaa = brca_clinical.join(cv_test_predicted_df_ihc[cv_test_predicted_df_ihc['Subtype']=='ER+/HER2-'], how='inner')[sss>=s]
aaa['Subtype'] = 'Higher predicted risk'
ttt = brca_clinical.join(cv_test_predicted_df_ihc[cv_test_predicted_df_ihc['Subtype']=='TNBC'], how='inner')
at = pd.concat([aaa,ttt])
result = km.fit(at[f'OS_MONTHS'], at[f'OS_STATUS'], at['Subtype'])
#     if result['logrank_P']<0.05:
km.plot(result, title=f"OS of ER+/HER2- with higher predicted risk VS TNBC, Logrank p-value={result['logrank_P']:.3e}", full_ylim=True, y_percentage=True)
plt.show() #Figure 4g
```
